# Multiscale Simulations of Self-Assembling Peptides: Surface and Core Hydrophobicity Determine Fibril Stability and Amyloid Aggregation

**DOI:** 10.1021/acs.biomac.4c00151

**Published:** 2024-04-23

**Authors:** Aysenur Iscen, Kübra Kaygisiz, Christopher V. Synatschke, Tanja Weil, Kurt Kremer

**Affiliations:** † Department of Polymer Theory, Max Planck Institute for Polymer Research, Ackermannweg 10, 55128 Mainz, Germany; ‡ Department of Synthesis of Macromolecules, Max Planck Institute for Polymer Research, Ackermannweg 10, 55128 Mainz, Germany

## Abstract

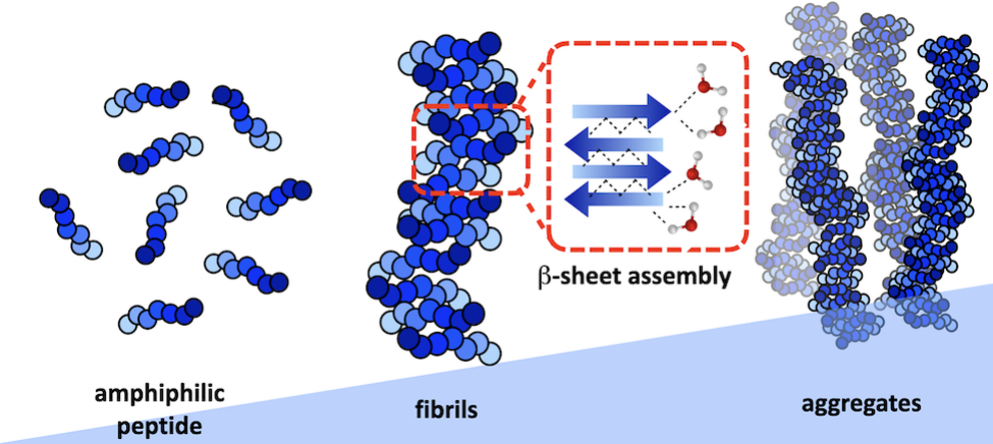

Assemblies of peptides and proteins through specific intermolecular interactions set the basis for macroscopic materials found in nature. Peptides provide easily tunable hydrogen-bonding interactions, which can lead to the formation of ordered structures such as highly stable β-sheets that can form amyloid-like supramolecular peptide nanofibrils (PNFs). PNFs are of special interest, as they could be considered as mimics of various fibrillar structures found in nature. In their ability to serve as supramolecular scaffolds, they could mimic certain features of the extracellular matrix to provide stability, interact with pathogens such as virions, and transduce signals between the outside and inside of cells. Many PNFs have been reported that reveal rich bioactivities. PNFs supporting neuronal cell growth or lentiviral gene transduction have been studied systematically, and their material properties were correlated to bioactivities. However, the impact of the structure of PNFs, their dynamics, and stabilities on their unique functions is still elusive. Herein, we provide a microscopic view of the self-assembled PNFs to unravel how the amino acid sequence of self-assembling peptides affects their secondary structure and dynamic properties of the peptides within supramolecular fibrils. Based on sequence truncation, amino acid substitution, and sequence reordering, we demonstrate that peptide–peptide aggregation propensity is critical to form bioactive β-sheet-rich structures. In contrast to previous studies, a very high peptide aggregation propensity reduces bioactivity due to intermolecular misalignment and instabilities that emerge when fibrils are in close proximity to other fibrils in solution. Our multiscale simulation approach correlates changes in biological activity back to single amino acid modifications. Understanding these relationships could lead to future material discoveries where the molecular sequence predictably determines the macroscopic properties and biological activity. In addition, our studies may provide new insights into naturally occurring amyloid fibrils in neurodegenerative diseases.

## Introduction

Nature has evolved β-sheet-rich, self-assembling peptide nanofibrils as functional scaffolds with inherent bioactivity. One of the most prominent examples of β-sheet-forming peptides is the structural class of β-amyloids.^1^ Amyloid peptide nanofibrils (PNFs) are characterized by their extraordinarily high stability and mechanical stiffness in the range of 2–3 GPa,^2^ which can be traced back to the distinct intermolecular hydrophobic interactions and hydrogen bonds.^3^

For decades, amyloid structures have been studied in the context of misfolded proteins, e.g., in neurodegenerative diseases.^4−6^ However, nonpathological amyloids also exist in nature for hormone storage^7^ or melanin polymerization^8^ in cells. In recent years, there has been an increasing interest in amyloids as functional materials to control bacteria biofilms,^9,10^ for capturing carbon from the environment^11,12^ and as therapeutic biomaterials such as enhancers of viral gene delivery^13−15^ and for neuron regeneration.^16,17^

These applications have also allowed deeper insights into the unique structural features that render certain amyloids functional. In particular, their interactions with cell membranes and virions have been studied in greater depth. It has been shown that positively charged amino acids (i.e., lysine) on the PNF surface enhance their binding to cells and viruses due to electrostatic interactions with the negatively charged cell and viral membranes, bringing them in close proximity, which boosts virus cell uptake and gene transfer. Depending on the intended application of PNFs, different functions of PNF may take effect, making it more complex to establish conclusive links between structure and properties. Therefore, strategies that combine advanced computational methods with experimental techniques are needed to gain more detailed insights into the structure and dynamics of this important class of biomaterials.

Amyloid formation is governed by a nucleation–elongation process, in which monomeric peptides first form oligomeric species consisting of β-sheets, which subsequently elongate into fibrillar structures.^18^ Differences in the assembly kinetics give rise to different lag times, which are of particular interest for the investigation of protein misfolding in amyloid structures.^19^ In fact, it has been hypothesized that pathological and functional amyloids differ from each other in their assembly kinetics that depend on the intermolecular interactions at the given conditions.^20−22^ Several decades of extensive research^23^ recognized that peptide–peptide interactions for initial amyloid formation are mainly driven by hydrophobic and electrostatic interactions.^24−27^ However, comparably less is known about the fibril–fibril interactions that determine the microscopic amyloid aggregation process. Although there is no conclusive evidence yet, it is thought that certain fibril surface properties, such as hydrophobic interactions,^28−30^ ion salt bridges,^25^ and polar interactions^31,32^ contribute to the aggregation of fibrils into larger structures.^31,33−35^ For example, fibrils with a hydrophobic surface expel water from the interface and prefer to interact with other hydrophobic fibrils.^33,34^ However, fibrils with polar surface groups can form hydrogen bonds between polar amino acids displayed at the surface, which also promotes aggregation.^31,35^ In the latter case, the environmental conditions, e.g., buffer salts, can suppress hydrophilic interactions in water, which then reduces fibril–fibril interactions.^31^

Molecular simulations are a powerful tool to shed light on the complex mechanisms that govern peptide self-assembly at experimentally inaccessible time and length scales. Previously, the structure of PNFs, which are composed of specific peptide sequences that have been shown experimentally to form β-sheet networks, has been studied using molecular dynamics simulations based on atomistic models.^36,37^ Moreover, coarse-grained models have also been used to investigate the self-assembly mechanisms of peptides into PNFs.^38−41^ Because each of these studies considers only a few specific peptide sequences, it is challenging to draw general conclusions about the structure–property relationships of PNFs. To explore the potential space of amino acid sequences for pentapeptide aggregation, a machine learning-based strategy was utilized where an artificial intelligence expert system performed a search to identify pentapeptides based on their aggregation propensities calculated from coarse-grained molecular dynamics simulations and compared to sequences suggested by human experts in the field.^42^ In this case, the scoring system for their model relied only on the hydrophobicity and aggregation propensity of amino acids and did not distinguish between different morphologies such as micelles, fibrils, vesicles, or sheets. Therefore, a systematic investigation of the impact of amino acid sequence on fibril morphology is still elusive.

Herein, we demonstrate that amino acid side chain hydrophobicity and position are critical to correlated morphology and biological properties of self-assembling PNFs over multiple length scales by applying molecular dynamics simulations. An amyloid peptide library that was tested for retroviral gene transfer^13,14^ was subjected to coarse-grained Martini^43^ model and atomistic CHARMM36^44^ force fields. In this way, we determined which amino acid sequences were more likely to assemble into supramolecular fibrils with high β-sheet contents in solution. We found that the dynamics of peptides within the amyloid fibrils are a critical parameter for bioactivity. Peptides with a low aggregation tendency are less likely to aggregate with other fibrils in solution, and the peptides in the supramolecular fibrils appear to be more mobile. In contrast, peptide sequences that form fibrils with a high content of β-sheets can interact with other fibrils, directly affecting the dynamics of the peptide monomers in the fibrils and making them less mobile and more active for retroviral gene transfer. These microscopic insights from a molecular design perspective to macroscopic features such as morphology, aggregation, and biological activities could pave the way to future materials discoveries. Moreover, we anticipate that the aggregation mechanism and material dynamics are underestimated, but potentially critical parameters that may also play an important role in naturally occurring amyloids and their occurrence in various fatal diseases.

## Computational Methods

Because self-assembly may take a long time (ns to μs), we performed coarse-grained (CG) simulations starting from randomly distributed peptides in solutions using three different concentrations: low concentration (40 peptides/1000 nm^3^ or 0.04 peptides/nm^3^), intermediate concentration (80 peptides/1000 nm^3^ or 0.08 peptides/nm^3^) and high concentration (20 peptides/125 nm^3^ or 0.16 peptides/nm^3^). The high-concentration simulations were designed with fewer peptides in a smaller simulation box in order to force the formation of a single fiber instead of aggregated structures.

After coarse-grained self-assembly simulations, we converted or “backmapped” our coarse-grained structures to all-atom (AA) model to gain additional information that can only be obtained from atomistic models, such as peptide secondary structure (β-sheet, helix, random coil), hydrogen bonding between peptides, hydrogen bonding with water, water configurations at the peptide surface, etc. When structures obtained from coarse-grained simulations are backmapped, sometimes the packing of molecules may not be perfect due to either low precision of interactions between molecules or time constraints. Therefore, we also performed atomistic simulations of perfectly aligned, preformed peptide nanofibrils for comparison. This latter approach is useful in order to give us some ideas about ideal packing situations, which may exist in experiments, and is a good way to interpret the results of coarse-grained simulations, where peptides are allowed to self-assemble in solution without any preconceived notion about what the structures may look like.

### Coarse-Grained Simulations

We performed molecular dynamics (MD) simulations of the self-assembly of peptides. For each peptide sequence, we used Packmol^45^ to randomly place peptides in a box for three peptide concentrations (0.04, 0.08, and 0.16 peptides/nm^3^). The coarse-grained peptides were modeled with the MARTINI force field version 2.2.^43^ Each simulation box was solvated using the polarizable MARTINI water model.^46^ We also added 0.138 M NaCl to mimic experiments and additional counterions (Cl^–^) to balance the total charge in the system. All coarse-grained simulations were performed with Gromacs 2019.4^47,48^ software according to the following protocol. We first minimized for 10,000 steps using steepest descent method. Then, we ran a constant volume (NVT) simulation for 1 ns using a 10 fs time step at 298 K in order to equilibrate the temperature. In the second step of the equilibration, we ran a constant pressure (NPT) simulation with a 20 fs time step for 2 μs at 298 K and 1 bar pressure to equilibrate the volume of the box. Following the equilibration, we performed a production run for 5 μs using the NPT ensemble with a 20 fs time step for the low and intermediate concentration simulations and 15 μs for the high-concentration simulations. We used the v-rescale coupling method^49^ for temperature and Parrinello–Rahman barostat^50,51^ for pressure coupling. The compressibility for the pressure coupling was 3 × 10^–4^ bar^–1^. Bond constraints were handled using the LINear Constraint Solver (LINCS) algorithm.^52^ The Verlet cutoff scheme^53^ was used for neighbor searching. Long-range electrostatics were treated with the reaction field method. A cutoff of 1.1 nm was used for the evaluation of all nonbonded interactions. For electrostatic interactions, we used the group method with a dielectric constant of 2.5, which is appropriate for the polarizable Martini water model. The neighbor list was updated every 10 steps. Atomic coordinates were saved every 500 ps for trajectory analysis. Periodic boundary conditions were set in *x*, *y*, and *z* directions. All snapshots from simulations were rendered using the Visual Molecular Dynamics (VMD) software.^54^ For backmapping of CG simulations to the atomistic model, see the Supporting Information.

We also performed simulations, where four self-assembled fibers from high-concentration CG simulations were placed in a box at a close distance as shown in Figure S1. The initial box size along the length of the fiber (*z*) was taken directly from the high-concentration self-assembly simulations (∼5 nm), and the box size for the other two dimensions (*x*, *y*) was set to 10 nm. There were a total of 80 peptides (20 × 4) in each box. The simulation boxes were solvated and ionized as before. The same equilibration protocol was used for these simulations, but the total simulation time was 10 μs.

### Atomistic Simulations of Preformed Fibers

In order to investigate the properties of perfectly aligned fibers, we performed MD simulations of preformed fibers. Since we know from experiments that these peptides self-assemble into Amyloid-like fibers with high β-sheet content, our initial structure was designed in order to optimize intermolecular hydrogen bonding between peptides by stacking 10 peptides along the length of the fiber with a peptide–peptide center of mass distance of 0.45 nm. This allows for a close packing of peptides that allows for intermolecular hydrogen bonding. From our previous CG self-assembly simulations, we observed that almost all peptides assembled to form dimer structures, where the hydrophobic residues of two peptides face each other while hydrophilic residues remain exposed to the surface of the fiber. Therefore, we placed another 10 peptides stacked in the same position across from the first layer to form a dimer, making sure that the charged lysine residues remain on the surface of the fiber. This preformed fiber structure was modeled with CHARMM36 force field^44^ and solvated with TIP3P water^55^ and 0.138 M NaCl as described in the previous section. The same MD settings were used for the equilibration and production runs. As discussed in the above section, the box volume was equilibrated first with isotropic coupling, followed by semi-isotropic coupling using the Parrinello–Rahman barostat.^50,51^ The total simulation run for each peptide sequence was 300 ns with a 2 fs time step. A summary of all simulations performed in this study is shown in Table S1.

## Results

**Figure 1:**
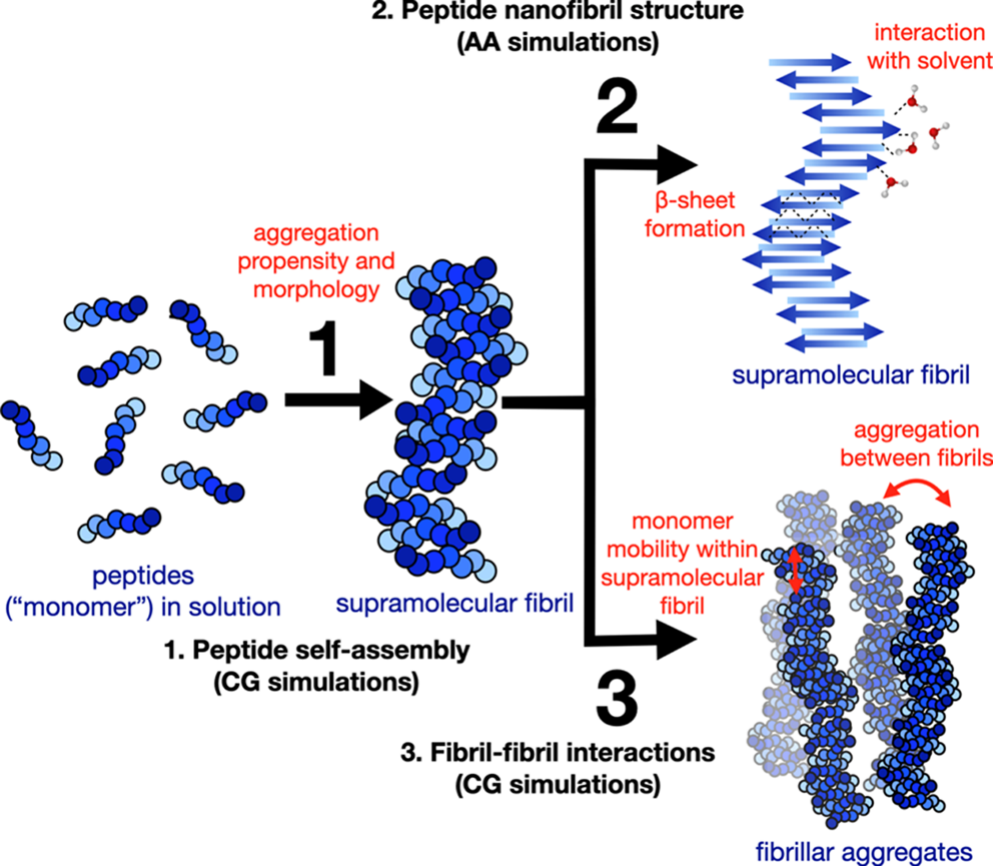
Summary of different steps, shown with black arrows, in multiscale simulations for understanding peptide aggregation in PNFs. Coarse-grained (CG) and all-atom (AA) representations of peptides are used for investigating different properties, shown in red. Peptides (“monomers”) in solution are illustrated as a sequence of beads in CG simulations. In the AA model, the directionality of monomers in the self-assembled supramolecular fibril is shown with arrows.

In this study, we aim to investigate how specific amino acid mutations can lead to amyloid formation by analyzing structure-morphology-property relationships in short self-assembling peptides from a molecular simulation perspective. For both naturally occurring amyloids and synthetic PNFs, the most important factor for their stability in solution is the formation of β-sheet structures. The concerted action of the hydrogen bond network provides long-term stability of the PNFs. Previously, the formation of β-sheets has been connected to the rich biological activity of functional PNFs.^13,14,16^ In this study, we investigate the structure formation of PNF across length scales, and identify monomer dynamics in PNFs and PNF aggregation as critical parameters for bioactivity. Figure 1 shows the different simulation strategies we employ in order to study the aggregation of peptides, i.e., monomers, into supramolecular fibrillar aggregates. We obtain various properties of monomers, supramolecular fibrils, and fibrillar aggregates from coarse-grained and all-atom simulations.

### Modeling Self-Assembly of Amyloid-like PNFs: Formation of β-Sheet Structures

We first studied the self-assembly process and the resulting fibril morphologies of two amphiphilic peptides that are known to form bioactive PNFs in solution. These studies are important for determining the simulation parameters, such as the force field, peptide monomer concentration, and simulation time. In order to investigate peptide self-assembly over several length scales, coarse-grained and fully atomistic simulations were performed. For these molecular simulations, we selected the peptide sequence CKIKQIINMWQ that forms long, thin fibrils with β-sheet structures in solution, which was published previously by us,^13,14^ as well as the short, β-sheet-forming region VHDCVNITIK (residues 176–185) of the human prion protein (PrP) that naturally forms amyloid fibrils. Misfolding of the PrP and accumulation in the brain is responsible for an infectious fatal neurodegenerative disease called “Prion disease” in humans and many animals.^56,57^ VHDCVNITIK and CKIKQIINMWQ peptides form nanofibrils with a similar β-sheet content and both sequences enhance virus transduction, although PNFs composed of CKIKQIINMWQ reveal higher bioactivity (Figure S3).

**Figure 2:**
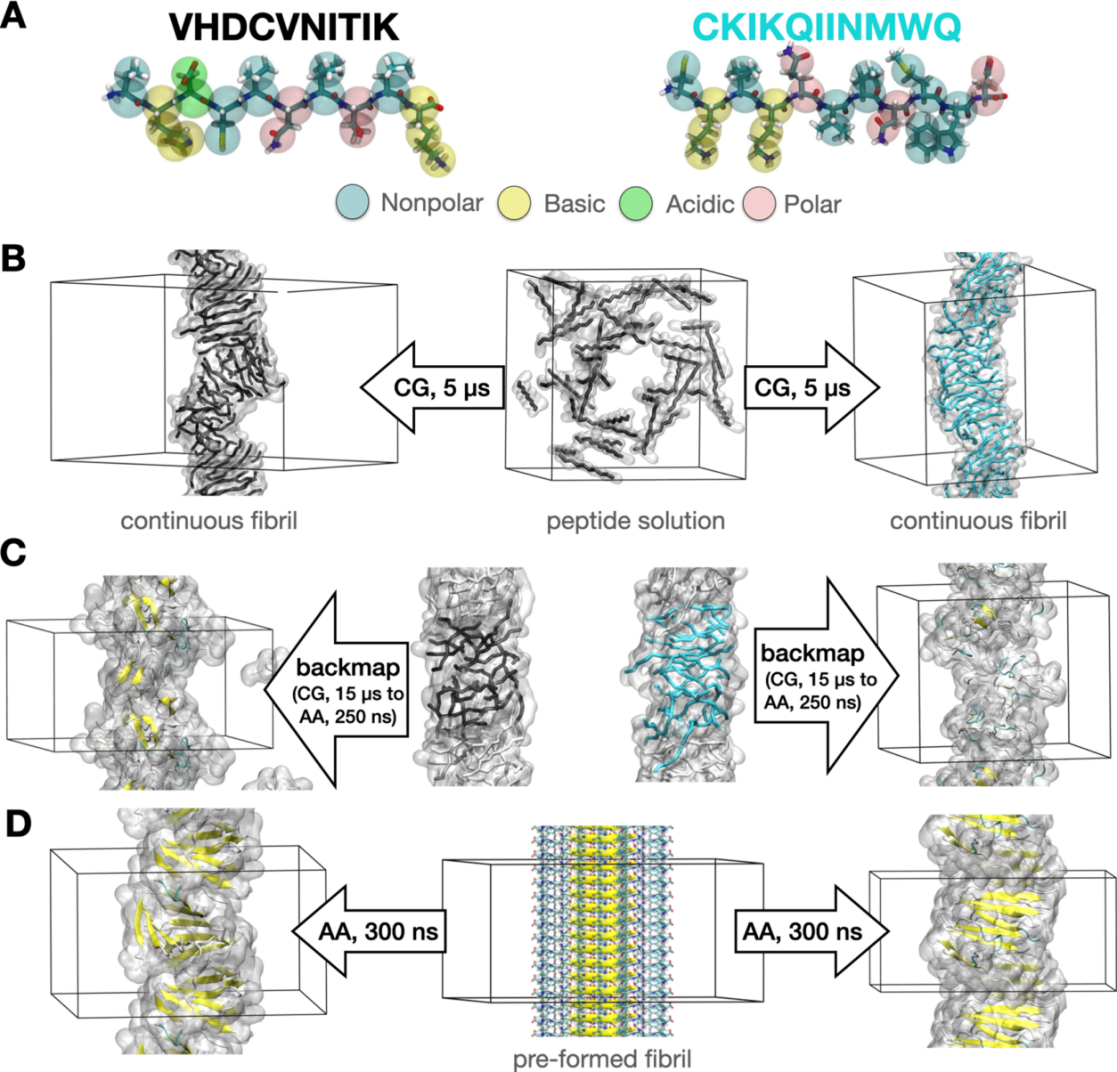
Multiscale model and different types of simulations performed for each peptide sequence in this study. (A) The coarse-grained model is used in simulations for the known peptide sequences VHDCVNITIK and CKIKQIINMWQ. Here, the beads are colored according to the amino acid side chain hydrophobicity and charges, which are coarse-grained into four categories (nonpolar, basic, acidic, and polar). (B) Coarse-grained (CG) self-assembly simulations at low peptide concentrations (0.04 peptides/nm^3^). Randomly distributed peptides in solution self-assemble to form PNFs that are extended over the periodic boundaries of the simulation box. (C) Coarse-grained self-assembly simulations at high peptide concentrations and the corresponding all-atom representation (AA) after backmapping and equilibration. (D) Atomistic simulations of the initial arrangement of preformed fibril and equilibration. Total simulation times for each simulation are given in the arrows. Peptide backbones are colored black for VHDCVNITIK (left panels, A–D) and blue for CKIKQIINMWQ (right panels, A–D) in the coarse-grained representation. In the atomistic representation, peptide backbones are drawn and colored according to the secondary structure, where β-sheets are shown in yellow. The rest of the peptide is shown in white. Water and ions have been omitted for the sake of clarity.

In the coarse-grained simulations depicted in Figure 2, the peptide monomers are randomly placed in a simulation box, and they are allowed to self-assemble over time. Self-assembly of low peptide concentrations (0.04 peptides/nm^3^) resulted in the formation of ordered PNFs shown in Figure 2B. The alignment of both peptides is approximately perpendicular to the long axis of the fibril along which the self-assembled structure is continuous over the periodic boundaries of the simulation box. The morphology of the fibrils shows twisted areas, where peptides are arranged in a slightly disordered fashion. However, once formed, these structures remain stable for the entire simulation time (5 μs) as shown in Movie S1. In the next step, we increase the monomer concentrations in our simulations. At intermediate concentrations, we observe the formation of larger aggregated structures with fibril-like morphologies, in which the peptides are aligned more or less perpendicular to the long fibril axis (Figure S4). The assembled fibrils appear thicker, and the twisting is more pronounced for these structures due to the higher peptide concentration. Both peptides self-assemble into a single PNF structure in less than 100 ns for low monomer concentrations and less than 5 ns for intermediate concentrations (Figure S3A). In the more highly concentrated solution, hydrophobic contacts between the lipophilic regions of the peptide amphiphiles occur more often, which accelerates the aggregation process. This result is in good agreement with earlier work by Fu et al.,^40^ who demonstrated that increasing the hydrophobic interactions of the amino acids of peptide amphiphiles results in the formation of more fibrillar morphologies.

The secondary structure in the coarse-grained model is fixed at the start of the simulation, and this method cannot provide secondary structure information i.e., the formation of β-sheets, α-helix, or random coil structures. Therefore, the secondary structure of both peptides is calculated using atomistic models. This approach is computationally expensive for low or intermediate peptide concentrations. Thus, we performed additional coarse-grained simulations at a higher concentration of 20 peptides/125 nm^3^ (0.16 peptides/nm^3^), shown in Figure 2C. We then converted the coarse-grained single fibril structures into an atomistic model using a backmapping procedure (see the Methods section in the Supporting Information). For both peptide sequences, the formed PNFs remain stable after conversion to the atomistic model and equilibration for 250 ns.

The mechanism of amyloid aggregation is complex and is not well understood. Simple coarse-grained models, such as the Martini model, do not allow folding of the peptide. Therefore, changes in the secondary structure cannot be obtained via this method and ordered amyloid structures that are observed in cryo-EM might not form in this model.^57^ During backmapping, it is particularly challenging to restore extended structures, which lead to β-sheet formation between peptides because the backbone beads in the Martini model are placed at the center of mass of each residue instead of the C_α_ position. Moreover, the backmapping procedure lacks the information to build the correct peptide planes that would result in extended regions in the atomistic model. Therefore, we constructed an amyloid fibril, shown in Figure 2D, from VHDCVNITIK and CKIKQIINMWQ using an atomistic model and tested the stability and β-sheet formation. Over the course of 300 ns simulation time, the preformed PNFs of CKIKQIINMWQ and VHDCVNITIK remain stable and the amount of β-sheet structures in the PNFs does not change (Figure S6C).

### Surface Hydrophobicity at the N-Terminus Enhances β-Sheet Formation and Fibril Stability

**Figure 3:**
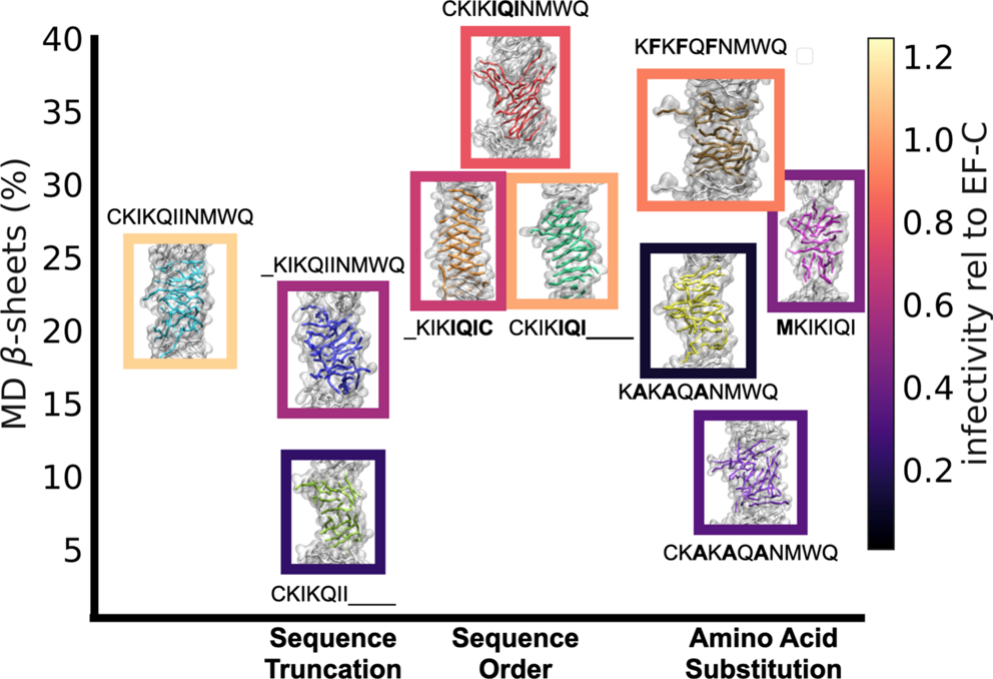
Effects of various chemical modifications, such as sequence truncation, change in sequence order, and amino acid substitution, on the peptide sequence on the relationship between intermolecular β-sheets and the biological activity of PNFs in retroviral transduction experiments. For each peptide sequence, snapshots of the self-assembled PNFs from high-concentration coarse-grained MD simulations are shown. Peptide backbones in the simulation box are colored according to the peptide sequence shown in the legend. The borders around each snapshot are colored according to the experimental infectivity measurements relative to CKIKQIINMWQ (EF-C).

In our previous work, the CKIKQIINMWQ peptide sequence has been used for the rational design of short amphiphilic self-assembling sequences, and their ability to self-assemble in solution has been quantified by determining the monomer to fibril conversion rates. Moreover, the surface charges and β-sheet structures have been acquired and structure-morphology-activity relationships have been established.^13^ We selected a subset of these peptide sequences, summarized in Table 1, to study the role of certain regions of the peptide in its self-assembly behavior with a computational approach. We started with the CKIKQIINMWQ peptide, discussed in the previous section, as reference. Specifically, we investigated the effect of sequence truncation (KIKQIINMWQ and CKIKQII), change in sequence order (CKIKIQINMWQ, CKIKIQI, and KIKIQIC), and amino acid substitutions (MKIKIQI, CKAKAQANMWQ, KAKAQANMWQ, and KFKFQFNMWQ). These modifications on the peptide sequence were done in order to understand how surface and core hydrophobicities alter the stability and aggregation of peptide nanofibrils in solution. The resulting self-assembled fibril structures from simulations and corresponding experimental infectivity data are summarized in Figure 3.

**Table 1:** Measured Properties of Selected Peptide Sequences Obtained from Experiments^13^ and Simulations

				β-sheet^d^ (%)					
sequence	infectivity rel. to EF-C^a^	fibril formation^b^	conversion rate^c^ (%)	EXP	MD	total SASA^e^ (nm^2^)	AP^f^ score (single fibril)	multifibril RMSF^g^, CG (nm)	normalized fibril–fibril distance^h^
VHDCVNITIK	0.55	+	N/A	51	22.9	140.7	1.86	2.35	0.76
CKIKQIINMWQ	1.24	+	50	51	22.8	169.3	1.58	4.06	1.29
KIKQIINMWQ	0.35	+	90	41	20.1	145.8	1.55	3.67	1.20
CKIKQII	0.04	+	55	25	7.8	181.3	1.75	3.91	0.24
CKIKIQI	1.02	+	54	53	27.8	125.2	2.03	3.10	0.25
CKIKIQINMWQ	0.7	+	89	52	35.2	152.9	1.69	3.60	1.06
KIKIQIC	0.61	+	52	53	28.1	106.8	2.10	3.50	1.70
CKAKAQANMWQ	0.04	–	92	39	12.2	172.1	1.51	3.82	0.76
KAKAQANMWQ	0.01	–	56	43	22.4	138.1	1.34	4.03	1.36
KFKFQFNMWQ	0.76	+	96	43	33.2	154.1	2.70	2.70	0.81
MKIKIQI	0.11	+	51	32	25.9	136.5	1.72	3.31	0.20

a Infectivity of HIV-1 was studied via a luminescence assay for β-galactosidase, which was reported by TZM–bl cells upon infection. EF-C is the peptide sequence QCKIKQIINMWQ.

b Positive (+) indicates fibril formation, while negative (−) indicates no fibril formation as determined from TEM.

c Conversion rate of PNFs is measured in experiments for conversion of monomers (i.e., peptides) to fibrils in solution.^14^

d Percentage of β-sheets is determined from FTIR spectra in experiments (EXP). Self-assembled (backmapped from CG) and preformed fibril models are used to determine the β-sheet formation in high peptide concentration atomistic MD simulations.

e Total solvent accessible surface area (SASA) is calculated in atomistic simulations using a probe radius of 0.14 nm, roughly corresponding to radius of water.

f Aggregation propensity (AP) score is calculated as the ratio of initial SASA (*t* = 0) to SASA (*t*) averaged over the last 2 μs of simulation.

g Root-mean-square fluctuations of per residue in CG multifibril simulations averaged over the last 2 μs of simulation.

h Fibril–fibril distance is normalized by the average fibril diameter for each nanofibril determined from CG simulation.

According to experimental data, the presence of the N-terminal cysteine (C) promotes the formation of β-sheet structures and enhances transduction rates in PNFs (**C**KIKQIINMWQ, etc.).^13,14^ In order to elucidate the impact of the terminal cysteine residue in the PNFs, we performed simulations comparing the CKIKQIINMWQ peptide to that of KIKQIINMWQ, without the N-terminal cysteine. Qualitatively, the obtained coarse-grained self-assembled PNFs are very similar and both sequences self-assemble into amyloid-like structures (Figures 3 and S7). At high concentrations, fibrillar structures are formed, which remain stable when backmapped to the atomistic model (Figure S8).

In a practical experiment, the time scales for self-assembly are much longer (∼min), and therefore, a variety of structures can form, including fibrils with defects and perfectly aligned fibrils. Therefore, in order to compare the assembly of two peptide sequences, in Figure 3 we use an average value of β-sheet content from both backmapped fibrils, where more defects are visible, and preformed fibrils consisting of more ordered assemblies. On average, the amount of β-sheets is about 20.1% for KIKQIINMWQ and 22.8% for CKIKQIINMWQ. This decrease in β-sheet formation is also observed in the experiments, where KIKQIINMWQ fibrils form PNFs with less β-sheet content (Table 1) suggesting that the N-terminal C may play an important role in structure formation and stability of the PNFs (Figure S8C). Interestingly, a loss in PNF stability is observed for simulations at low and intermediate monomer concentrations (Figure S7A,B), where KIKQIINMWQ PNF oligomers are observed. In contrast, CKIKQIINMWQ forms a continuous PNF under these conditions (Figures 2B and S4). Our model indicates a reduced β-sheet content and lower stability of the KIKQIINMWQ PNFs compared to CKIKQIINMWQ, which is also accompanied by low infectivity-enhancing features (Table 1).

**Figure 4:**
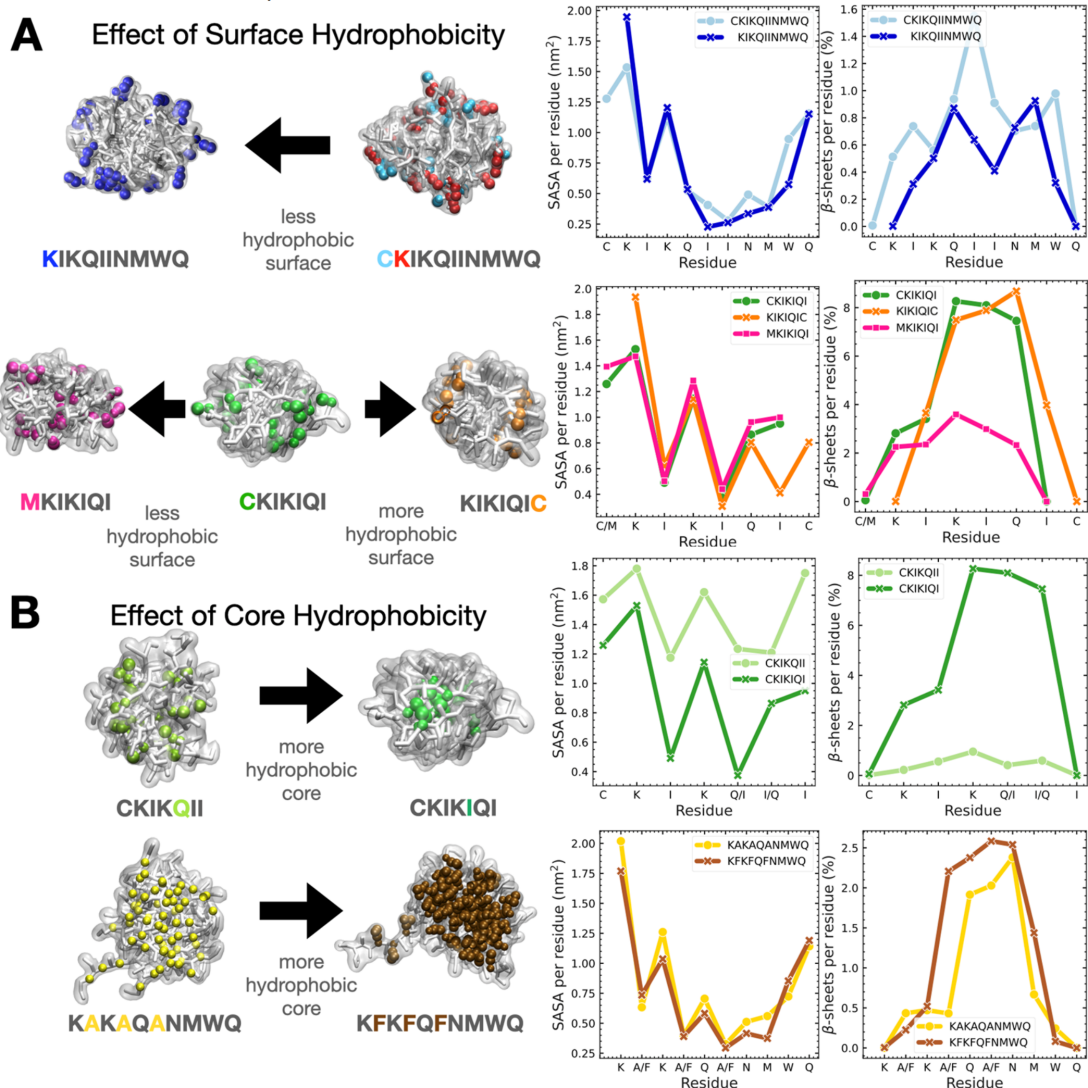
Effects of the (A) surface and (B) core hydrophobicity and amino acid distributions over the fibril cross section on β-sheet formation. Fibril cross-sectional (top) views are colored according to the highlighted amino acid residue in the peptide sequence. Average solvent accessible surface area (SASA) and β-sheets per residue are calculated from the last 2 μs of simulation. The percentage of β-sheets is calculated per total number of residues in the simulation box.

The slower self-assembly kinetics of **K**IKQIINMWQ vs **C**KIKQIINMWQ could be attributed to the role of the N-terminal residue: According to the solvent accessible surface area (SASA) calculations in Figure 4A, the N-terminal cysteine efficiently shields the rest of the peptide in the PNFs from water molecules and ions, promoting peptide–peptide interactions. In contrast, the surface hydrophobicity of **K**IKQIINMWQ PNFs reveals a significant increase in peptide-solvent interactions at the N-terminus, shown in Figure 4A, which is based on the charged side chain of this lysine residue that is fully exposed to the solvent and is exclusively located at the surface of the **K**IKQIINMWQ PNFs. In contrast, the first cysteine residue reduces the contact area of the second lysine (residue 2) with solvent molecules in the **CK**IKQIINMWQ PNFs. As a consequence, the amino acids close to the ends of both peptides interact more with solvent molecules, and they are less likely to form β-sheet structures (Figure 4A). Therefore, the presence of the hydrophobic N-terminal cysteine residues increases self-assembly kinetics and promotes the formation of stable PNFs with high β-sheet content.

Next, we studied the influence of the cysteine residue located at the C-terminus. We have compared the short peptide sequences with N-terminal cysteine **C**KIKIQI and with C-terminal cysteine KIKIQI**C** (Figures 3 and S12). Experiments show that switching the position of the N-terminal cysteine to the C-terminus has a significant effect on the formed structures (Table 1). Even though CKIKIQI and KIKIQIC form PNFs with similar β-sheet contents (Figure 3), the resulting structures are remarkably different. The cross section of the **C**KIKIQI fibril reveals less ordered stacking of the peptides along the length of the fibril. In contrast, KIKIQI**C** peptides form flat, ribbon-like fibrils, where the peptides are perfectly aligned so that the hydrophobic amino acids are buried and the lysine residues are exposed at the surface (Figure 4A). The main difference between these structures is how the two ends of the peptide chains interact with the water molecules. Without cysteine, the N-terminus of the KIKIQI**C** sequence is highly hydrophilic and the C-terminal cysteine is less exposed to the surface and interacts less with solvent molecules compared to the N-terminal cysteine (Figure 4A). In Figure 4, we found very similar β-sheet self-assembly behavior, but in the active fibrils (**C**KIKIQI), the cysteine residues are arranged on the fibril surface more evenly rather than being concentrated on two ends. We think this could enhance fibril–fibril interactions in solution and support the formation of hierarchical structures, which will be discussed later. These differences could result in remarkably different infection rates, which are much lower when cysteine is located on the C-terminus (Table 1).

In order to further elucidate the specific role of the N-terminal cysteine, this residue was replaced with a more hydrophobic methionine. At high concentrations, **M**KIKIQI assembles into less ordered fibrils compared to **C**KIKIQI (Figures 3 and S17). While some of the peptides of **M**KIKIQI are aligned perpendicular to the long axis of the fibril, others are oriented in different directions, which leads to a more random distribution of the methionine over the cross-sectional area of the fibril (Figure 4A). While cysteine is more concentrated on the surface of the **C**KIKIQI fibrils, some of the methionine residues are also buried close to the core of the MKIKIQI fibril. This has direct consequences in terms of the peptide–peptide interactions at the atomistic level. The bulky side chain of methionine interrupts the hydrogen-bonding interactions between those peptides that are involved in β-sheet formation. Consequently, **M**KIKIQI forms considerably less β-sheet structures in the core region in contrast to **C**KIKIQI fibrils with a very high amount of these ordered structures (Figure 4A). Although methionine is more hydrophobic compared to cysteine in solution, the higher disorder and the occurrence of defects near the core of the fibril make the surface of the **M**KIKIQI fibrils more hydrophilic. These fibrils also reveal no enhancement in transduction efficiency^11^ (Table 1), which seems to be connected with higher surface hydrophilicity and less ordered assemblies.

On the other end of the CKIKQIINMWQ peptide, the NMWQ sequence was initially thought to enhance transduction in PNFs as the removal of NMWQ from CKIKQIINMWQ peptide resulted in a drastic decrease in biological activity (CKIKQII, Table 1). However, after testing other sequences, it was found that the motif NMWQ was not essential for enhancing the retroviral transduction properties.^11^ Our simulations show that when NMWQ sequence is removed from the CKIKQIINMWQ peptide, CKIKQII peptides form disordered aggregates at low and intermediate concentrations instead of a fibril (Figure S19). A fibrillar structure forms only at high concentration (Figure 3 and Movie S2), but this structure is not stable and dissociates completely when backmapped to an atomistic representation (Figure S20). In both experiments and simulations, there is a major loss of β-sheet structures for CKIKQII fibrils (Table 1 and Figure 4B). Nevertheless, this does not mean that all short peptide sequences perform poorly. A single change in the order of amino acids, i.e., CKIKQII to CKIKIQI, enhances the stability and β-sheet propensity of peptides, as well as its transduction enhancement, which will be discussed in the next section.

### Fibril Core Hydrophobicity Increases Order and β-Sheet Formation

So far, we have shown that the stability and formation of β-sheet structures in supramolecular nanofibrils can be enhanced by increasing the peptide–peptide interaction and minimizing the peptide–water interaction. This can be done by modification of the ends of the peptide chains via the presence of a hydrophobic amino acid, such as cysteine, or optimization of the internal amphiphilic pattern. In this section, we explore how the latter approach can tune the properties of peptide nanofibrils by the substitution of amino acids in the core region.

#### Effect of Amphiphilic Pattern

In order to optimize the internal packing of the peptides, Sieste et al.^14^ proposed an intrinsic switch of fifth and sixth residues (CKIK**QII** to CKIK**IQI**), which produces an alternating hydrophilic–hydrophobic pattern of the amino acids. The CKIK**IQI** sequence has one of the highest percentages of β-sheets (27.8%, Table 1 and Figure 3) even though it lacks the NMWQ motif and the fibers do not dissociate in simulations over time as in the case of CKIK**QII**. We also observed the same effect, although not as pronounced, in the longer peptide sequences CKIK**QII**NMWQ and CKIK**IQI**NMWQ. Since the presence of the NMWQ fragment already increases stability, the stabilizing effect of **IQI** sequence in CKIK**IQI**NMWQ is less pronounced compared to the short peptide sequences that lack the NMWQ fragment (Figure S21), which is in agreement with Sieste et al.^14^

The importance of a single switch in the amino acid sequence in terms of the fibril properties is best explained through its interactions with solvent molecules. Figure 4B shows the spatial distribution of the fifth residue, where the switch happens, and its interaction with water in the self-assembled fibril structures. The fifth residue in the peptide sequence is important since it is preceded by a pattern of basic–hydrophobic–basic (–KIK–) amino acids, where Q residues are distributed closer to the fibril surface, and I residues are located at the core region. In other words, having a hydrophobic amino acid near the core of the peptide allows for better packing and increases peptide–peptide interactions that lead to the formation of β-sheets.

#### Isoleucine to Alanine/Phenylalanine Substitution

Experiments show that alanine-containing PNFs have very low amounts of β-sheet formation and infectivity in retroviral transduction. The self-assembled CKAKAQANMWQ fibril shows some β-sheet formation (12.2%, Table 1 and Figure 3), but the fibrillar structure did not remain stable in atomistic simulations (Figure S26). Moreover, while preformed CKIKIQINMWQ fibril revealed remarkable stability and a large amount of β-sheet structures, CKAKAQANMWQ peptides were more disordered and we observed a decrease in β-sheets over time as well as the formation of 3_10_-helix structures (Figure S18E–G). This is in good agreement with the experimentally determined propensity of amino acids to form helix structures, in which alanine has the highest propensity compared to all other amino acids.^58^

On the other hand, replacing alanine with a more hydrophobic amino acid, such as phenylalanine, results in the formation of fibrils with high β-sheet content (33.2%, Table 1 and Figure 3) even without the N-terminal cysteine present. KFKFQFNMWQ peptide sequence forms continuous fibrils for all concentrations tested in our simulations (Figures S27 and S28). This observation agrees well with the TEM images (Figure S27E), where KAKAQANMWQ self-assembles into aggregates while KFKFQFNMWQ produces long, thin fibrils. The aggregation of the hydrophobic phenylalanine residues in the core of the fibril (Figure 4B) enables less peptide-water interactions of the fibril core and water/ions, which results in the formation of a more stable, β-sheet-rich core.

### Fibril–Fibril Interactions Promote Hierarchical PNF Assembly and Bioactivity

We have simulated how peptides self-assemble in solution, their secondary structure formation, and their stability in solution. However, in these simulations, only the formation of a single fibril is considered due to the finite size of the simulation boxes. In contrast, TEM images often show the formation of fibril aggregates (Figure S3D). It is conceivable that the surface properties such as surface polarity of PNFs could influence PNF solvent and fibril–fibril interactions.^10^ In principle, the self-assembly process could continue after the formation of single fibrils and could also lead to the formation of hierarchical structures. The dynamic features of amyloid-like PNFs are still not well understood, and experimental methods to study the monomer dynamics in PNFs are lacking. Therefore, we studied the formation of higher-order PNF assemblies by applying coarse-grained simulations of interacting fibrils in solution. By considering the dynamic properties of PNFs, i.e., the interactions and dynamics of peptide monomers in the PNFs, we could gain deeper insights into the interaction of monomers within PNFs, their diffusion within the PNFs as well as their exchange between different PNFs and the ability to form new PNFs in solution.

Experimental results of TEM, light scattering, and wide-field microscopy measurements can image peptide aggregation and structure formation over multiple length scales. According to these experimental data, some peptide sequences such as CKIK**QII** form well-dispersed single PNFs, whereas others, such as CKIK**IQI**, tend to aggregate in solution to form μm-sized aggregates.^13^ In order to understand the structural origins of these experimentally observed differences in PNF assembly, in our simulations, we placed four fibrils, self-assembled at high peptide concentration, and in very close proximity (Figures 6 and S1). In this way, not only can the peptides in each supramolecular fibril interact through “intra-fibril” interactions, but the monomers in the PNFs can also “sense” the presence of peptide monomers in the other fibrils through “interfibril” interactions. After 10 μs of simulation of the PNFs for each peptide sequence, we observed three distinct behaviors: (I) The PNFs remain stable, well hydrated, and isolated in solution. (II) PNFs remain stable but interactions of different PNFs occur, whereas in (III), PNFs lose stability, remix, and form new aggregates and/or fibrils in solution.

**Figure 5:**
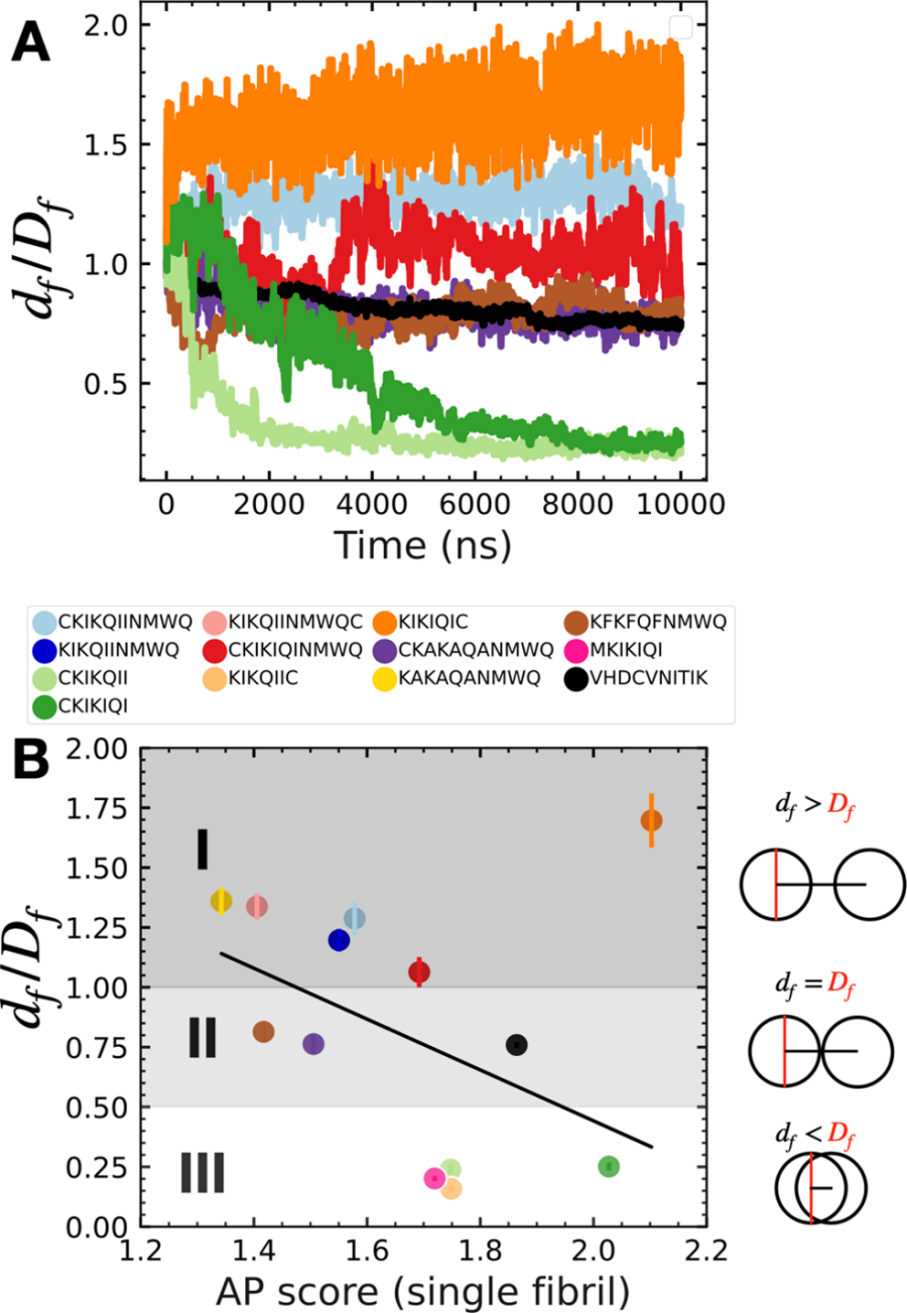
(A) Selected examples for the average fibril–fibril distance (*d*_f_) over time from coarse-grained simulations of four interacting fibrils normalized by fibril diameter (*D*_f_). A large value indicates minimal or no interfibril interaction, while a value close to 0 indicates remixing of peptides to form new structures. (B) Relationship between peptide aggregation propensity (AP) score from single fibril self-assembly simulations and fibril–fibril distance from multifibril simulations. Three regions are shown for no fibril–fibril aggregation (I), moderate fibril–fibril aggregation (II), and large fibril–fibril aggregation where fibrils lose stability and remix to form new fibrils (III).

Because the peptides have a different number of amino acids, it is challenging to compare the assembled structures based on a single property such as the surface area. Therefore, we use the distance between the center of mass of fibrils in solution, normalized by the diameter of a single fibril for each peptide sequence, to distinguish the differences in the aggregation behavior of the fibrils, shown in Figure 5. Rapidly decreasing fibril–fibril distances indicate that the fibrils lose stability and remix in solution (III), while a slight decrease in the fibril distance refers to those fibrils that remain stable but interact with other fibrils to form aggregated fibril bundles (II). On the other hand, an increase in fibril–fibril distance refers to fibrils that remain stable and noninteracting in solution (I). Representative examples of these three different PNFs are shown in Figure 5A. Interestingly, peptide sequences that lack the C-terminal NMWQ fragment dissociate and remix. Obviously, the NMWQ sequence improves the PNF stability in solution. One exception is the sequence KIKIQIC that remains stable and noninteracting in solution. This behavior could be explained by the high surface hydrophilicity as depicted in Figure 4A.

Next, we investigate whether there is a direct relationship between the aggregation of fibrils in solution and the ability of peptides to self-assemble into a single fibril. When the peptides are randomly dispersed in solution (Figure 2B), the total surface area of the peptides exposed to the solvent molecules is high. Over time, peptides self-assemble into PNFs due to van der Waals interactions as well as hydrogen bonds. This process reduces the solvent accessible surface area (SASA) of the peptides. We calculated an aggregation propensity (AP) score as the ratio of SASA (*t* = 0) to SASA (*t*). A larger value of the AP score refers to a greater tendency of peptides to self-assemble into PNFs.

Our simulations reveal that the tendency for peptide assembly, expressed by the AP, could be linked to reduced fibril–fibril distances, as shown by multiple interacting fibrils in solution, depicted in Figure 5B. This means that peptides with a high aggregation propensity continue to interact favorably even after self-assembly into supramolecular fibrils and lead to fibril–fibril aggregation. VHDCVNITIK has a high AP score of 1.86 and self-assembles into fibrils that are more likely to interact and aggregate in solution. Shorter peptide sequence such as CKIKIQI, lacking the NMWQ fragment, also reveal high AP values above 1.7, but the formed fibrils do not remain stable in solution. In contrast, the peptide monomers of different fibrils will mix when they move closely to form new fibrils. The outlier in Figure 5B, i.e., KIKIQIC, has a high AP score with no fibril–fibril aggregation due to the arrangement of cysteine and lysine groups on the surface (Figures 4B and S31). However, even for similar AP scores, the self-assembling properties of peptides in solution can vary significantly when the fibrils are in contact with each other depending on the hydrophobicity and interaction of the amino acids on the PNF surface. Thus, the peptide AP score describing the self-assembly of a single fibril has to be combined with simulation data of multiple fibrils in solution to validate the formation of hierarchical structures.

**Figure 6:**
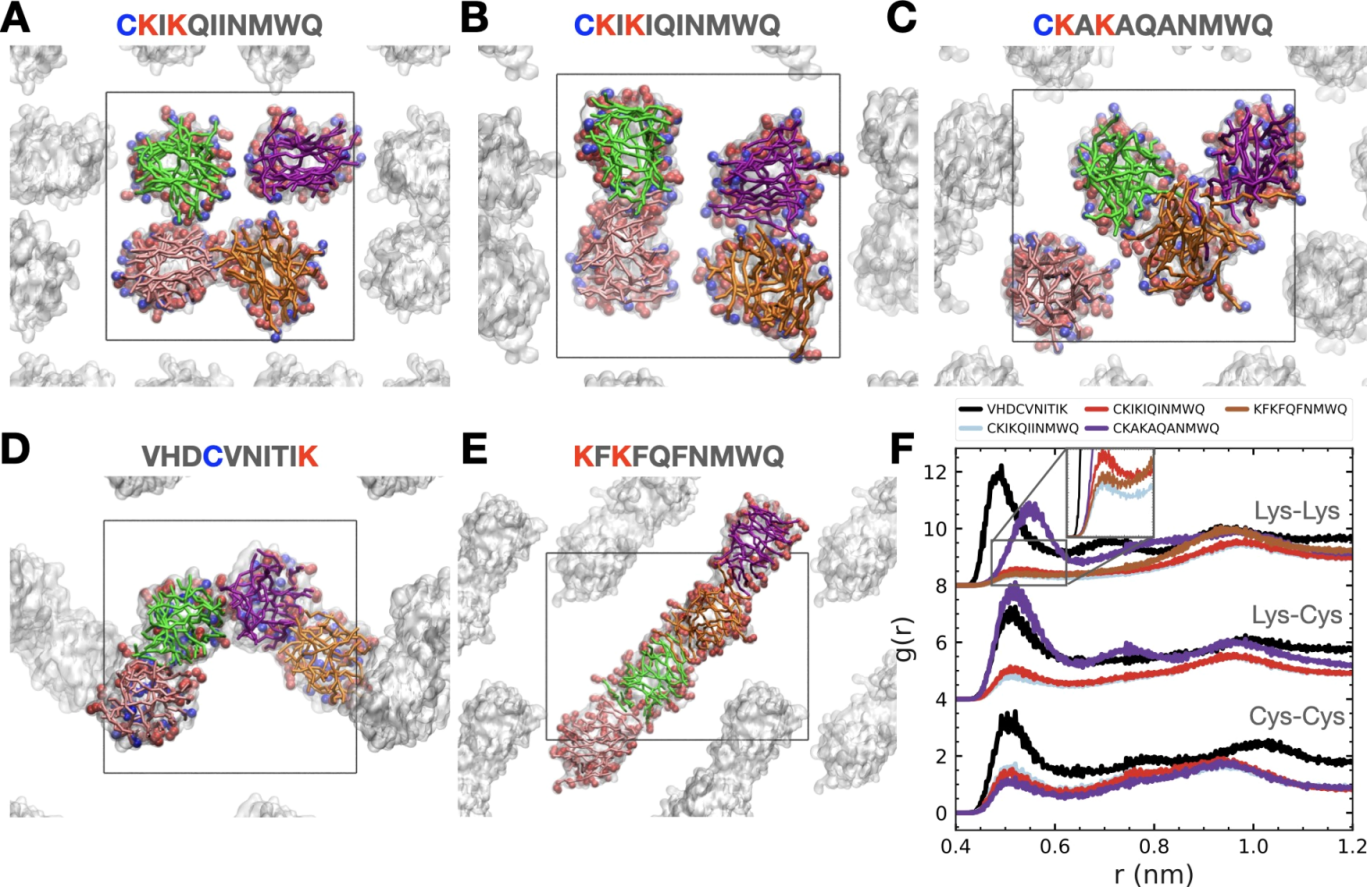
Cross-sectional views from 10 μs of simulations of interacting fibrils that remain stable in solution for (A) CKIKQIINMWQ, (B) CKIKIQINMWQ, (C) CKAKAQANMWQ, (D) VHDCVNITIK, and (E) KFKFQFNMWQ. Here, the backbones of peptides that belong to the same fibril in the initial structure (see Figure S1) are colored with the same color. Cysteine residues are colored blue, and lysine residues are red. The size of the simulation box is colored black to show the interaction over periodic images. The side chain beads of the peptide are colored white. Water and ions have been omitted for the sake of clarity. (F) Intermolecular pair distribution functions, *g*(*r*), between cysteine and lysine residues. The *g*(*r*) values for lysine–cysteine and lysine–lysine are shifted for easier viewing.

In Figure 6, we show selected examples of fibrils that remain stable in solution but display varying degrees of interactions. According to the cross-sectional views of the snapshots, CKIKQIINMWQ fibrils that do not aggregate in solution still reveal fibril–fibril interactions that keep the fibrils in close proximity. For the fibrils consisting of the optimized CKIKIQINMWQ sequence, a slight decrease in the fibril–fibril distance is observed, indicating slightly stronger interactions between the fibrils.

In our simulations, the interactions between the fibrils proceed through the displacement of water molecules between fibril surfaces. Thus, formed structures closely resemble cross β-spine structures found in amyloid-like fibrils,^31^ where a stack of steric zipper structures forms the dry interface between the β-sheets and different fibrils interact in solution through the wet interface.

When the hydrophobic isoleucine residues are replaced with alanine, CKAKAQANMWQ fibrils also remain stable and aggregate in solution (Figure 6C). However, when investigated closely, we observe some mixing of peptides within the aggregated fibril bundle (purple and green peptides in the orange fibril). This means that the fibrils tend to lose stability due to peptide remixing, similar to the cases observed with short sequences. This observation further confirms our previous discussion on how replacing isoleucine with less hydrophobic alanine decreases the stability of the PNFs and makes them more prone to forming aggregates in solution rather than fibrils.

A very favorable interfibril interaction exists for the VHDCVNITIK sequence. Based on the cross-sectional view, most of the cysteine residues are buried in the core of the fibrils. The single lysine residue located at the C-terminus of the peptide chain provides a positively charged side chain close to the negatively charged (−COO^–^) backbone. This colocalization results in more favorable interactions, stabilizing the fibril surfaces, which increases the interactions between the fibrils in solution (Figure 6D). Another example of a peptide sequence that displays a strong interfibril interaction is KFKFQFNMWQ. Here, the resulting aggregated fibril structure is different compared to VHDCVNITIK, where the fibrils align in a way that maximizes their contact while maintaining lysine-rich surfaces on each side. Remarkably, this aggregated fibril morphology resembles the morphologies that were extracted in Cryo-EM for a similar phenylalanine containing PNF (CKFKFQF), where individual fibrils stacked together over time to form thicker morphologies with multiple hydrophobic and polar contact modes.^32^

Recent experiments^59^ have shown evidence of disulfide bridge formation between the N-terminal cysteine residues in self-assembled fibrils. Because the disulfide bridge is a chemical bond that forms between the sulfur atoms of two cysteine residues, we are not able to observe it in our coarse-grained simulations. However, for this reaction to happen, the side chains of cysteine residues must be very close to each other in solution, and we can quantify the distribution of cysteine and positively charged lysine residues by calculating intermolecular pair distribution functions, *g*(*r*), shown in Figure 6F. Since the VHDCVNITIK fibrils are closest in solution, the magnitude of the first peak for lysine–lysine, cysteine–lysine, and cysteine–cysteine distributions are very large compared to other structures in Figure 6. CKAKAQANMWQ also aggregates in solution, but while lysine–lysine contacts are strong, cysteine–cysteine interaction is the weakest among the compared structures. This means disulfide bridge formation is very unlikely in CKAKAQANMWQ fibrils. After VHDCVNITIK, CKIKQIINMWQ shows the most favorable cysteine–cysteine interaction followed by CKIKIQINMWQ. Lysine–lysine interactions follow an opposite trend for these structures, with more closely distributed lysine residues in CKIKIQINMWQ. What is interesting is that even though the KFKFQFNMWQ fibrils are highly aggregated in solution, the lysine–lysine contacts are small due to their orientation with respect to each other.

## Discussion

**Figure 7:**
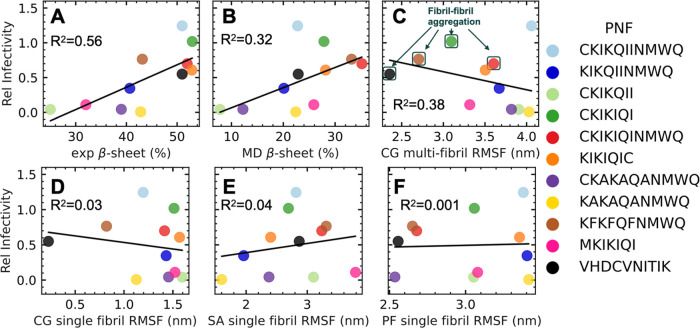
Correlation between different measured properties and relative infectivity. While we observe a correlation for the data in the top row (A–C), there is no direct link between properties shown in the bottom row (D–F) and infectivity. Here, SA and PF single fibril simulations refer to self-assembled (backmapped) and preformed atomistic simulations of single fibrils in solution.

Our simulations and experiments show that the primary structure (amino acid sequence) of a peptide is an important factor in determining the morphology of self-assembled structures, whether it forms a long amyloid-like fibril or only small aggregates in solution. Although experiment and simulation conditions, such as the peptide concentration or the type of model used, can lead to differences in the observed structures, we can learn from the trends in our data to derive structure–property relationships. Figure 7 shows how properties correlate with the infectivity rates for the subset of peptide sequences that we have chosen for our simulations. Because both experimental (Figure 7A) and MD (Figure 7B) β-sheet amounts depend on conditions, such as the peptide concentration, here we compare the trends in our data instead of the absolute quantity. Overall, there is remarkable agreement between the simulations and experiments, where (1) the presence of N-terminal cysteine increases β-sheet formation for isoleucine-containing peptides (KIKQIINMWQ vs CKIKQIINMWQ), but not for alanine-containing peptides (KAKAQANMWQ vs CKAKAQANMWQ); (2) switching cysteine position from N-terminal to C-terminal does not affect the β-sheet amount (CKIKIQI vs KIKIQIC); (3) removing the C-terminal NMWQ eliminates the formation of β-sheets in the original sequence (CKIKQIINMWQ vs CKIKQII); (4) optimization of the amphiphilic pattern induces β-sheet formation even for short sequences (CKIKQII vs CKIKIQI); (5) replacing the N-terminal cysteine with more hydrophobic methionine causes loss of β-sheet-rich structure (CKIKIQI vs MKIKIQI); and finally (6) increasing the core hydrophobicity of internal residues by replacing alanine with phenylalanine greatly improves the β-sheet formation (KAKAQANMWQ vs KFKFQFNMWQ).

While the dynamic nature of these supramolecular peptide nanofibrils is one of the properties that sets these materials apart from conventional polymers, so far, it is not clear how this dynamic property affects their function. Thus, we also investigated whether the peptide mobility affects the biological activity of the PNFs. As previously discussed, the number of β-sheets from both experiments and simulations is positively correlated to infection rates in cells. We also find that there is a weak negative correlation between root-mean-square fluctuations (RMSF) of peptides and infectivity in multifibril simulations where there is fibril–fibril interaction, as shown in Figure 7C. The RMSF is providing information on the mobility of peptide monomers in a fibril. We notice that CKIKQIINMWQ peptides (outlier in Figure 7C) have high RMSF in fibrillar form because they do not aggregate in our simulations but also perform very efficiently in experiments, which may be due to the high experimental β-sheet content of these fibrils that is not reproduced in simulations. In fact, if CKIKQIINMWQ is omitted, the correlation between MD calculated β-sheets and relative infectivity (Figure 7B) improves to 0.53, similar to the correlation between experimental β-sheets and relative infectivity (Figure 7A). Our results suggest aggregation of fibrillar structures reduces the mobility of peptide chains (low RMSF) in the supramolecular fibril and formation of hierarchical structures through fibril–fibril aggregation (low RMSF) correlates positively with bioactivity (Figure 7C). Interestingly, this correlation between monomer mobility and bioactivity is only observed in multifiber simulations and not in single-fiber atomistic or coarse-grained simulations (Figure 7D–F). These results show that while single fibril simulations in solution are beneficial in providing structural properties, such as β-sheet formation, fibril–fibril interactions provide important information about the dynamical behavior of peptides and should be taken into account when structure-morphology-property relationships are investigated.

## Conclusions

Understanding structure–property relationships of supramolecular peptide nanofibrils is important for identifying amino acid sequences to facilitate the discovery of functional materials with optimized properties. Herein, we present firm evidence from multiscale modeling of how specific changes in the primary sequence of amphiphilic self-assembling peptides influence the unique features of self-assembled peptide nanostructures, their secondary structure formation, morphology, fibril stability, and fibril aggregation in solution. We combined coarse-grained and atomistic simulations to draw conclusions between the microscopic properties and biological activity of PNFs.

Our results show that sequence truncation reduces the stability of the supramolecular fibrils in solution, and shorter amphiphilic peptide sequences mostly afford the formation of stable oligomers. The presence of the C-terminal cysteine residue plays an important role, as it increases the hydrophobicity of the PNF surface by shielding the β-sheet-forming regions from water and ions, which leads to the formation of long and stable PNFs in solution. Removing the C-terminal cysteine reduces the number of β-sheet structures, which destabilizes the PNFs in solution. Furthermore, when the N-terminal NMWQ motif is missing, less stable fibers with a very low amount of β-sheets form, and the stability of fibrils can be recovered only with reordering of the internal amino acids to make an amphiphilic pattern. The introduction of amino acids with more hydrophobic side chains (Val, Leu, and Phe) improves the stability of fibrils when the substitution is done on the internal residues. However, a very hydrophobic C-terminus with high hydrophobicity disrupts the β-sheet-forming region.

Our studies also shed light on the dynamic properties of peptides within the supramolecular fibrils. While a high aggregation propensity of peptides leads to fast self-assembly in solution, it can also be detrimental to the stability of fibrils when in the proximity of other fibrils in solution. A low aggregation propensity results in the formation of no aggregated hierarchical structures, which is highly correlated with increased biological activity. Those peptide sequences with “moderate” aggregation propensity self-assemble into stable PNF that can further aggregate to multifibrillar assemblies in solution, revealing the strongest bioactivity in our assays.

Because these PNFs are inspired by Amyloid fibrils, we tested our model by comparing the self-assembly of our chosen peptide sequences to a short, β-sheet-forming region of human prion protein (VHDCVNITIK), which is one of the naturally occurring amyloid-forming proteins that accumulate in the brain leading to a fatal, neurodegenerative disease. Thus, we have shown that our multiscale approach is applicable for studying the properties of both synthetic and naturally occurring Amyloid-like peptide nanofibrils.

## Supporting Information Available

Stability of the formed structures over the entire simulation time (Movie S1) (MP4)
Formation of fibrillar structure at high concentrations (Movie S2) (MP4)
Detailed description of the computational methods, table with details of simulations, additional simulation data, supporting figures, and discussion (PDF)

## Abbreviations

PNF: peptide nanofibril
MD: molecular dynamics
PrP: prion protein
AA: all-atom
CG: coarse-grained
TEM: transmission electron microscopy
EM: electron microscopy
EF-C: enhancing factor C
SASA: solvent accessible surface area
*D*_f_: fibril diameter
*d*_f_: fibril–fibril distance
AP: aggregation propensity
RMSF: root-mean-square fluctuations
SA: self-assembled
PF: preformed
