# Supplementary material for: Multiscale Simulations of Self-Assembling Peptides: Surface and Core Hydrophobicity Determine Fibril Stability and Amyloid Aggregation
Source: Biomacromolecules. 2024 Apr 23;25(5):3063–75. doi: 10.1021/acs.biomac.4c00151 (PMC11094720; doi:10.1021/acs.biomac.4c00151)
Supplement: Supplementary file 3 — bm4c00151_si_003.pdf [file bm4c00151_si_003.pdf]

Supporting Information: Multi-scale simulations of self-assembling peptides: surface and core hydrophobicity determine fibril stability and amyloid aggregation

Aysenur Iscen,* Kübra Kaygisiz, Christopher V. Synatschke, Tanja Weil, and Kurt Kremer*

Max Planck Institute for Polymer Research, Ackermannweg 10, 55128 Mainz, Germany

E-mail: iscena@mpip-mainz.mpg.de; kremer@mpip-mainz.mpg.de

Computational Methods

Coarse-grained simulations

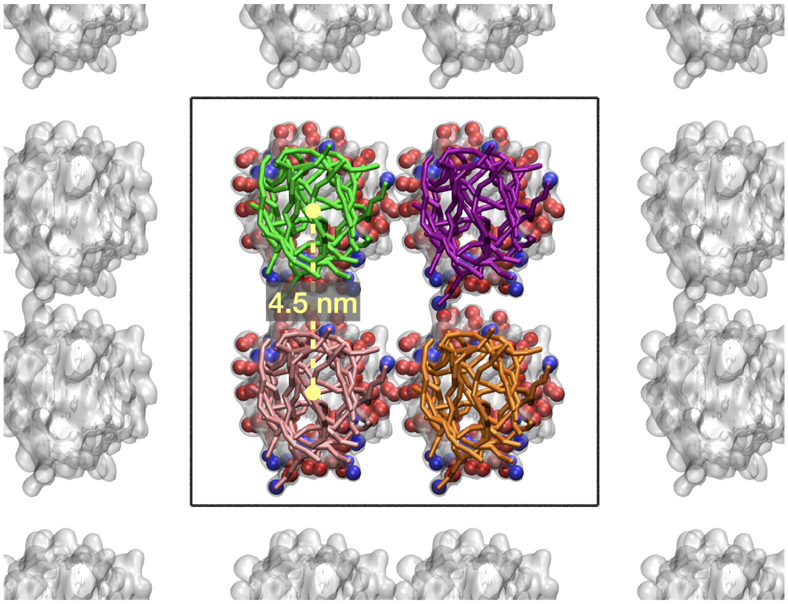

Figure S1: The cross-sectional view of initial configuration of four peptides placed with fiber-fiber center of mass distance of 4.5 nm. Peptides that belong to the same fiber have backbones colored green, purple, pink and orange. Cysteine residues are colored in blue, lysine is red. The side chains of peptides are shown in white. The simulation box boundaries are drawn in black. Water and ions are omitted for clarity.

Backmapping of CG to AA model

Due to limitations on system size and timescales associated with atomistic simulations, we only backmapped our high concentration CG simulations to atomistic model. This is useful for our analysis because due to high concentrations of peptides, we observe a single, self-assembled fiber for all peptide sequences at this concentration with the CG model. However, even at the CG level, some of these fibers are more stable than others. By converting our CG fibers to atomistic representation, we gain information on stability of the fibers in solution and secondary structure formation. For the backmapping procedure, we used our equilibrated single fibers (after 15 μs) as initial coordinates and converted them into

full atomistic resolution according to the CHARMM36[S1] force field. We minimized the backmapped peptides using two steps of minimization: 1) 500 steps with steepest descent method where nonbonded interactions between peptides are excluded and 2) 500 steps with steepest descent method without any exclusions. After the short minimization, we solvated and ionized the atomistic fiber with TIP3P water[S2] and 0.138 M NaCl as before. After this step, following simulation protocol was used for equilibration of the atomistic structures. We first minimized the system for 1000 steps, followed by a short NVT equilibration at 298 K for 200 ps, where the peptide was position constrained. Following this, we performed an NPT equilibration at 298 K and 1 bar using isotropic pressure coupling for 100 ns. An addition production NPT simulation was performed for 150 ns at the same temperature and pressure, but using semiisotropic pressure coupling to allow for box size along the z direction to change independently from x and y. For atomistic simulations, the time step was 2 fs. Long-range electrostatics were determined with the smooth particle mesh Ewald (PME)[S3] method using cubic interpolation and Fourier grid spacing of 0.16 nm. Van der Waals interactions were smoothly switched to zero between 1.0 and 1.2 nm. A cut-off of 1.2 nm was used for evaluation of all non-bonded interactions. Atomic coordinates were saved every 100 ps for the trajectory analysis. The compressibility for the pressure coupling was 4.5 x 10^{-5} bar^{-1}. Only hydrogen bonds were constrained using the LINCS algorithm.[S4] Secondary structure analysis was performed using the STRIDE algorithm[S5] included in VMD.[S6]

Atomistic simulations of pre-formed fibers

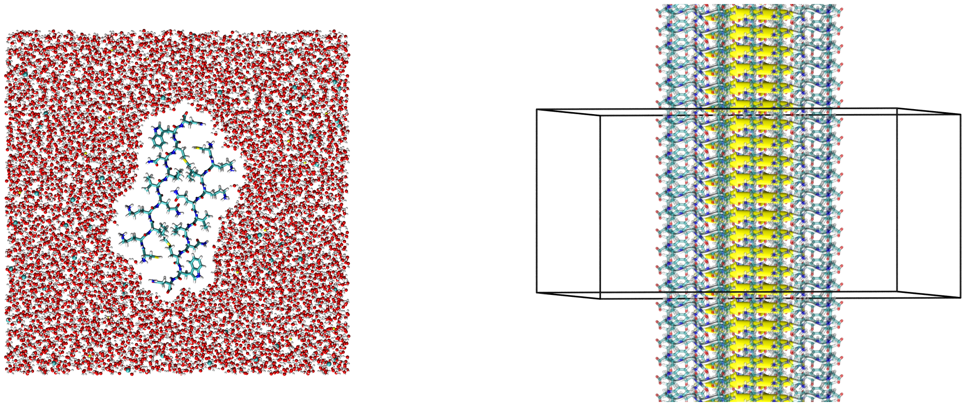

(a) Top view

(b) Side view

Figure S2: The initial (a) cross-sectional top and (b) side views of the pre-formed fibers. Along the cross-section of the fiber (top view), two peptide chains antiparallel to each other are placed so that their lysine residues are on the surface of the fiber, while hydrophobic isoleucine residues are buried in the core. Along the length of the fiber (side view), ten peptides are placed with an initial center of mass distance of 4.5 nm. There are a total of 20 peptides in this system. Simulation box is shown in black. Peptides only interact with their periodic image along the length of the fiber in order to simulate an infinitely long fiber. Water and ions are omitted for clarity.

A summary of all simulations performed in this study is shown below in Table S1. We tested a total of 13 peptide sequences and performed 6 different simulations for each peptide sequence. Four of these simulations were coarse-grained simulations using the Martini model, and the other two were atomistic simulations using the CHARMM36 force field. The total simulation time for a single peptide sequence was 35,000 ns (35 µs) and 550 ns for coarse-grained and atomistic simulations, respectively. In total, we ran 455 µs of coarse-grained simulations and 7150 ns of atomistic simulations.

[1]The number of peptides for each simulation is same for all 13 peptide sequences studied. However, due to differences in the peptide sequences, the number of water and ions (total number of bead/atoms) slightly differ. The numbers given in the table refer to simulations with CKIKQIINMWQ peptide sequence.

Table S1: Summary of simulation details for different types of coarse-grained and atomistic simulations performed in this study.[1]

Peptide sequences	Force field	Type of simulation	Number of peptides	Number of water	Number of ions	Total number of beads/atoms	Simulation time (ns)
CKIKQIINMWQ, CKIKIQINMWQ, KIKQIINMWQ, KIKQIINMWQC, CKIKQII, CKIKIQI, MKIKIQI, KIKQIIC, KIKIQIC, CKAKAQANMWQ, KAKAQANMWQ, KFKFQFNMWQ, VHDCVNITIK	Coarse-grained Martini	Self-assembly, low concentration (0.066 M)	40	6992 (PW model)	83 Na, 163 Cl	22302	5,000
		Self-assembly, intermediate concentration (0.13 M)	80	2706 (PW model)	42 Na, 202 Cl	21968	5,000
		Self-assembly, high concentration (0.26 M)	20	628 (PW model)	10 Na, 50 Cl	2484	15,000
		Interacting fibers (4 self-assembled, high concentration fibers)	80	2563 (PW model)	42 Na, 202 Cl	10093	10,000
	All-atom CHARMM36	Self-assembled, high concentration backmapped from Martini	20	7138 (TIP3P model)	21 Na, 61 Cl	25576	250
		Pre-formed fiber	20	7433 (TIP3P model)	21 Na, 61 Cl	26461	300

Supporting Figures for Results Section

Modeling self-assembly of Amyloid-like PNFs: formation of β-sheet structures

One of the factors that significantly affect the morphology of the self-assembled structures is the peptide concentration both in experiments and simulations. Because we use periodic conditions in our simulations, depending on the peptide concentration, we can observe different morphologies such as aggregates or continuous fibrillar structures. Therefore, we consider

three different concentrations as discussed in the Methods section. Both VHDCVNITIK and CKIKQIINMWQ self-assemble to form fibers in low (0.04 peptides/nm^3) concentration, shown in Fig. 2 in main text.

In the next step, we keep the box size constant and double the number of peptides to investigate whether similar morphologies are available at intermediate (0.08 peptides/nm^3) concentration. As seen in the figure below, the peptides still self-assemble into fibers, however the fiber twists to accommodate the larger number of peptides.

In terms of the different peptide sequences, the VHDCVNITIK peptide self-assembles into a fiber more quickly than CKIKQIINMWQ peptide at low concentration. This difference in self-assembly kinetics can be attributed to the arrangement of amino acids in the sequence. Both peptides are of similar length (10 vs 11 total residues per peptide chain, respectively) and contain similar ratios of nonpolar (5/10 vs 6/11) and polar (2/10 vs 3/11) amino acids. However, the interaction of the peptides with water in assembled fibers is different. The solvent accessible surface area (SASA) of the amino acids in the assembled fiber structures is similar when we consider the two ends of the peptides (N- and C-terminus) as they contain charged amino acids at both ends that interact with water and ions in solution. However, in the case of CKIKQIINMWQ, the charged lysine groups are located in 2nd and 4th position, where the latter position is close to the β-sheet forming core region of the peptide. The effect of these lysine residues is clearly visible with high SASA value, whereas VHDCVNITIK fiber becomes more hydrophobic closer to the core region (Fig. S3B). The stronger hydrophobic interactions between the peptides increases the kinetics of self-assembly for VHDCVNITIK peptide compared to the CKIKQIINMWQ peptide.

We also consider a high peptide concentration (0.16 peptides/nm^3), but with a smaller box size and less number of peptides. This is done in order to investigate how a small section of the PNF looks like when it self-assembles. The morphologies for CKIKQIINMWQ and VHDCVNITIK are shown in Fig. 1C in the main text. On average, 16 % of VHDCVNITIK peptides form β-sheet structures, which is about twice the amount formed by

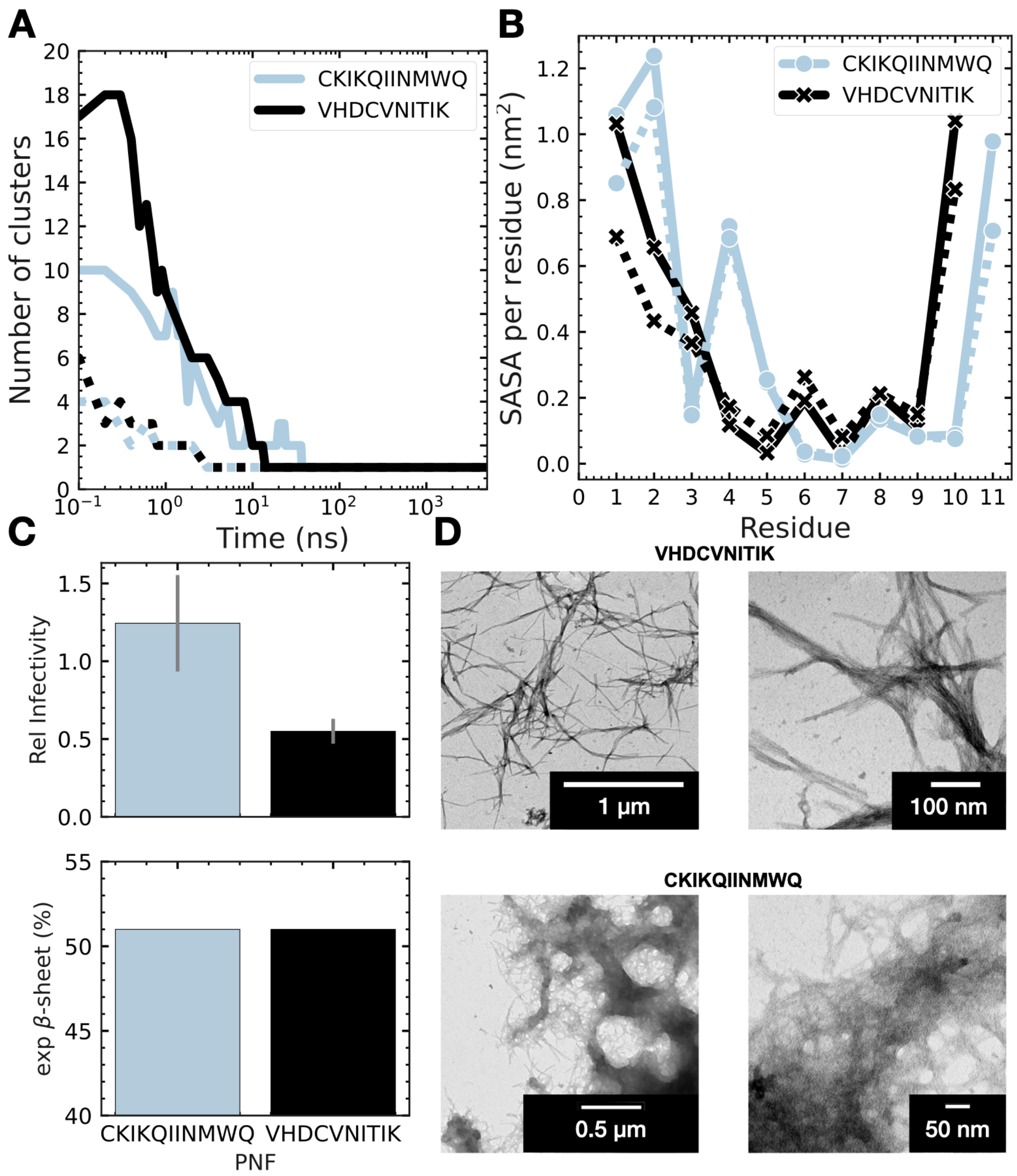

Figure S3: Comparisons between (A) self-assembly kinetics depicted by the change in number of clusters over simulation time and (B) average solvent accessible surface area (SASA) per residue from self-assembled CKIKQIINMWQ and VHDCVNITIK fibrils from the last 2 µs of simulation. The solid lines refer to simulations at low peptide concentration (shown in A and B), while dotted lines refer to simulations at intermediate peptide concentration (see Fig. S4). (C) Experimental data comparing relative infectivity at 1.3 µM concentrations and intermolecular β-sheet content of peptides. The error bars refer to standard deviation from different measurements. (D) TEM images for VHDCVNITIK and CKIKQIINMWQ fibers.

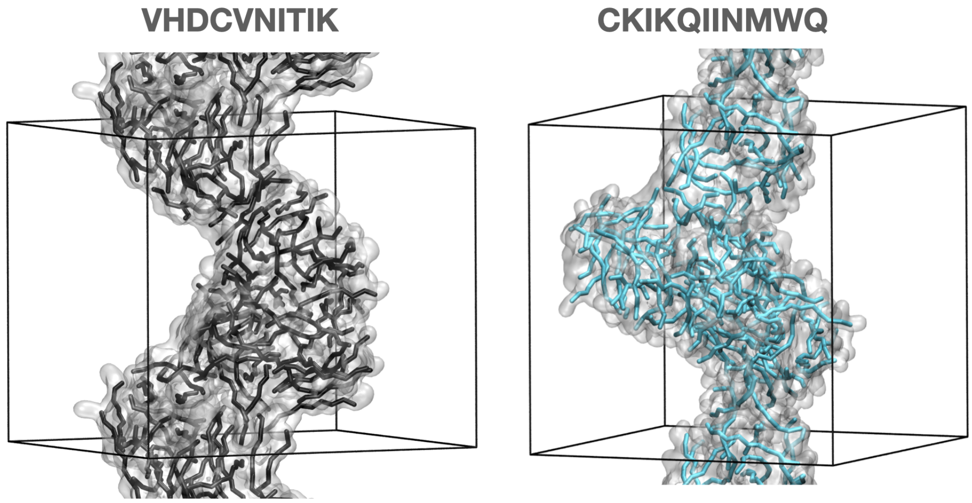

Figure S4: Snapshots from intermediate concentration (0.08 peptides/nm^3) coarse-grained self-assembly simulations after 5 μs. Only peptide backbones are colored for easier view of peptide assembly. The rest of the peptides are shown in white. Water and ions are omitted for clarity.

CKIKQIINMWQ peptides (7.8 %) (Fig. S5A). It's important to note here that the β-sheet content from backmapped atomistic structures is very small compared to experiments, which is due to the missing secondary structure information when backmapping from Martini to CHARMM model. During backmapping, it is especially difficult to restore extended structures, which lead to β-sheet formation between peptides, because the backbone beads in the Martini model are placed at the center of mass of each residue instead of C_α position, and the backmapping procedure lacks the information to build the correct peptide planes that would result in extended regions in atomistic model.[S7] Nevertheless, we use these backmapped atomistic structures to understand the trends in the experimental data and also to more accurately design the pre-formed fibers, which will be discussed later.

For the VHDCVNITIK peptide, there is an increase in the amount of β-sheets that each amino acid makes starting from the N-terminus that reaches a maximum with the 8th residue (threonine, T). Amino acids close to the two ends of the peptide chain are exposed to the water and ions, and the strong interactions with solution prevent them from forming hydrogen bonds with other peptides that are necessary for forming intermolecular

β-sheets structures. The number of β-sheets is strongly correlated with the solvent-accessible surface area of each amino acid in the assembled fiber (Fig. S5C). This explains the low amount of β-sheets observed in CKIKQIINMWQ fiber, where instead of a very hydrophobic core region as in VHDCVNITIK fiber, the 4th amino acid residue (lysine, K) is highly hydrophilic. This means that the lysine residues are on the surface of the fiber, where water and ions can penetrate and prevent peptide-peptide contacts. We should also mention that the SASA trends for each amino acid calculated from atomistic simulations at high concentration matches very well to the SASA calculated from coarse-grained simulations at low and intermediate concentrations, suggesting trends we observe in terms of how peptides self-assemble and interact with solvent are not dependent on peptide concentration or the computational model used.

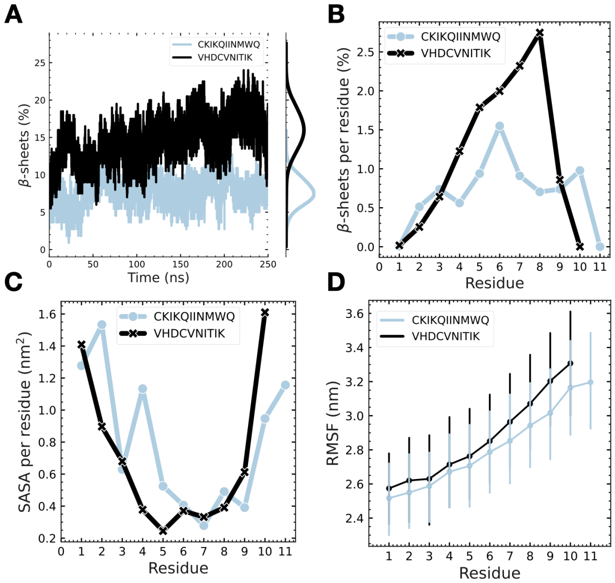

Figure S5: The percentage of (A) total β-sheets over time, (B) average β-sheets per residue, (C) average solvent accessible surface area (SASA) per residue and (D) average root mean square fluctuation per residue (RMSF) calculated from self-assembled atomistic fiber simulations after the backmapping procedure for CKIKQIINMWQ and VHDCVNITIK. In A and B, percentage is calculated per total number of residues in the simulation box. For B-D, only the last 100 ns of the simulation is used for calculating the time average values.

Recently, a study by Álvarez et al.[S8] showed that similar self-assembling peptides are effective in promoting corticospinal axon regrowth after acute spinal cord injury in mice.

However, surprisingly the authors of this study found that the disruption of the β-sheet region increases the efficiency of nanofibers in promoting neuronal differentiation of neural progenitor cells in vitro. Authors speculate that the added motion of disordered protein domains that results from disruption of β-sheet structures provides greater capacity to signal efficiently in the biological environment. Their coarse-grained model and simulations provide useful information about the mobility of peptides in assembled structures, but the authors do not extend their computational model to provide any information about secondary structures, i.e. β-sheet formation, in peptide nanofibers. Our simulations show that while the mobility of the peptides, which we determine by calculation of root mean square fluctuation of each residue, in self-assembled fibers is comparable for both peptides, VHDCVNITIK also has a higher percentage of β-sheets (Fig. S5B,D). Interestingly, while both fibers start with similar amounts of β-sheets structures after the backmapping procedure, VHDCVNITIK develops more β-sheets over 250 ns simulation time while CKIKQIINMWQ remains the same. This suggests that perhaps high mobility of peptides in the fibers does not necessarily mean loss of stability as suggested by Álvarez et al.[S8], but can also contribute to structure formation.

Although we are able to achieve fiber formation in all three concentrations for coarse-grained self-assembly simulations, we recognize that because these simulations are coarse-grained, the packing of the peptides may not be ideal or perfect as in a Amyloid formation. Therefore, we also tested the stability of a peptide fiber that is pre-formed based on prior knowledge of how peptides self-assemble in solution from CG simulations, but without the defects in the structure. Although CKIKQIINMWQ initially has more β-sheet structures compared to VHDCVNITIK, the fluctuations in number of β-sheets is also larger and at the end, both fibers have similar amount of β-sheets present.

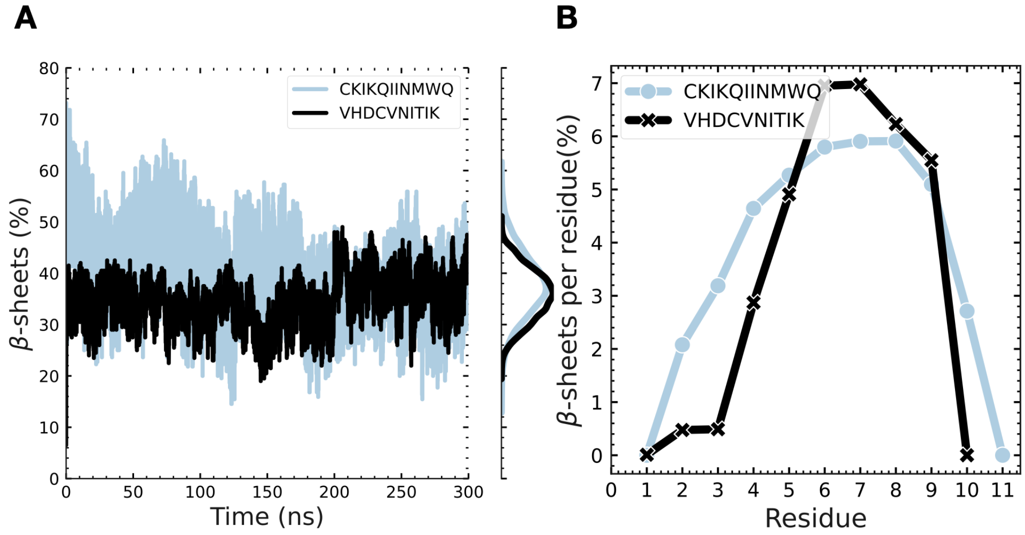

Figure S6: (A) Total percentage of amino acid residues making β-sheets structures over the simulation and (B) average β-sheet amount per residue for pre-formed CKIKQIINMWQ and VHDCVNITIK fibrils calculated from the last 100 ns of simulation. Both in A and B, percentage is calculate with respect to the total number of amino acid residues.

The pre-formed fibrils have higher β-sheet contents, because the peptides are packed together with minimal defect in the initial fiber structure and since it is fully atomistic, there is no backmapping procedure that may result in loss of structural information. While β-sheets in pre-formed VHDCVNITIK fiber is distributed in a very similar manner to the self-assembled fiber, where we observe the highest β-sheet content for residues 4-9, the trends observed for pre-formed CKIKQIINMWQ fiber is slightly different to its self-assembled counterpart. Pre-formed CKIKQIINMWQ fiber shows a steady increase in the number of β-sheets starting from the N-terminus and reaching a maximum at the 8th residue (asparagine, N) and slowly loses structure towards the C-terminal of the peptide chain. With perfectly aligned peptides, the effect of water-peptide interaction at the core region of the fiber is eliminated and strong peptide-peptide interactions provide increased stability against losing structure. This suggests that over time CKIKQIINMWQ peptides can form nanofibers with similar β-sheet content as those of VHDCVNITIK peptides, which explains the similarities between β-sheet content measured in experiments.

Surface hydrophobicity at the N-terminus enhances β-sheet formation and fibril stability

The structures from self-assembly simulations at low and intermediate concentrations are shown in the Figure below for KIKQIINMWQ peptide sequence. This peptide self-assembles to form a fiber-like segment at both concentrations, which is not continuous over any of the periodic boundaries of the simulation box. Surface hydrophobicity is very similar except for the N-terminus, where the cysteine amino acid is missing, making this fiber less hydrophobic. This enhanced interaction of the N-terminus with water prevents the peptide to form a continuous fiber for these two concentrations.

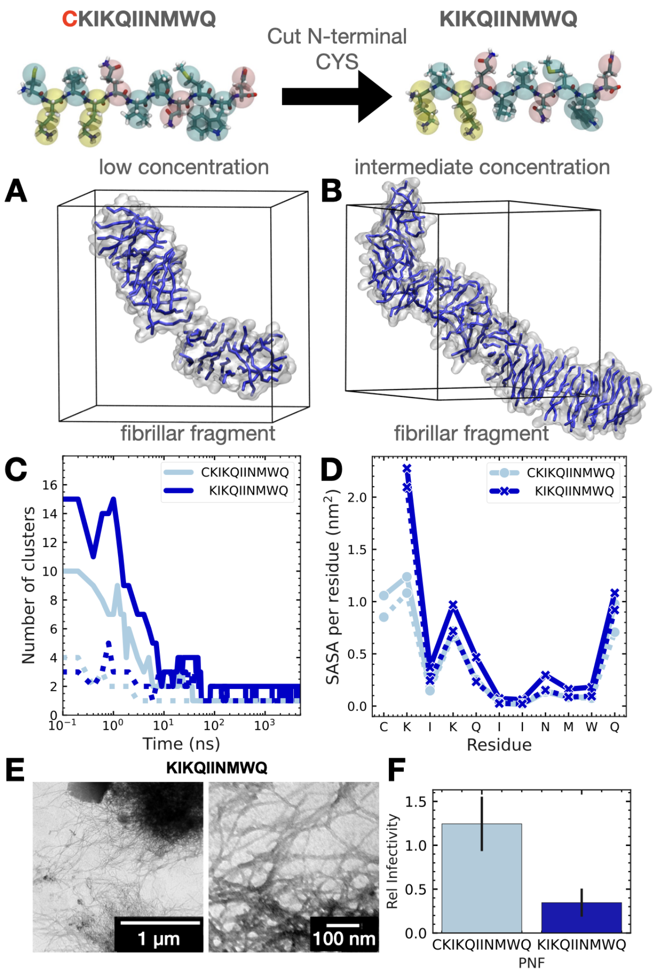

Figure S7: Snapshots from (A) low and (B) intermediate concentration coarse-grained self-assembly simulations after 5 μs for KIKQIINMWQ. Only peptide backbones are colored for easier view of peptide assembly. The side chains are shown in white. Water and ions are omitted for clarity. (C) Number of clusters during self-assembly as a function of simulation time. (D) Average solvent accessible surface area (SASA) per residue calculated from the last 2 μs of simulation. The solid lines refer to simulations at low peptide concentration, while dotted lines refer to simulations at intermediate peptide concentration. (E) TEM images for KIKQIINMWQ fibers. (F) Experimental infecivity relative to EF-C comparing CKIKQIINMWQ to KIKQIINMWQ.

Compared to CKIKQIINMWQ, the number of peptides that form β-sheets decreases upon removal of N-terminal cysteine. The decrease in β-sheet formation is also observed in experiments for KIKQIINMWQ fibers suggesting that N-terminal C may play a role in structure formation and stability of the PNFs. This loss in stability is observed in low and intermediate concentration simulations (Fig. S7), where the structures resemble fragment

of a fiber instead of a continuous one that we obtained earlier with CKIKQIINMWQ. At low concentration, absence of the N-terminal C leads peptides to aggregate slower and the formed structure fluctuates between one or two aggregates.

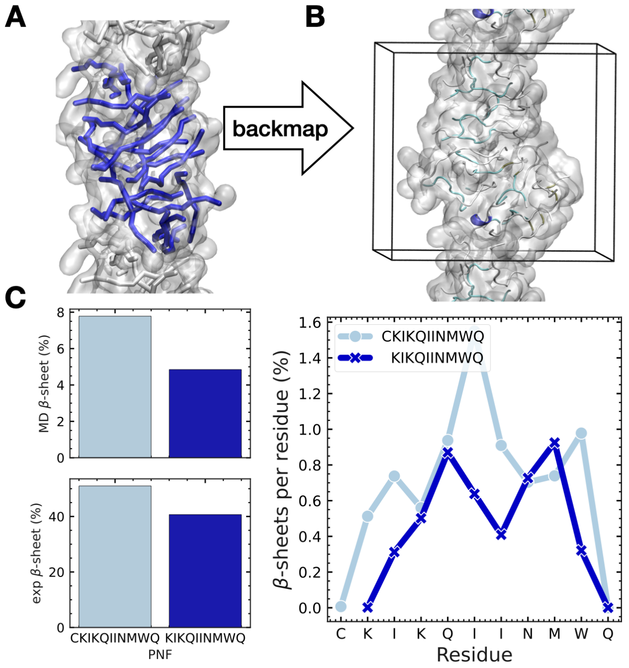

Figure S8: Self-assembled KIKQIINMWQ fiber at high peptide concentration from (A) coarse-grained and (B) backmapped atomistic simulations. (C) The change in the average β-sheet content with removal of N-terminal cysteine from experiments and MD simulations and the average percentage of β-sheets per residue.

Role of the N-terminal cysteine is further investigated by comparing CKAKAQANMWQ and KAKAQANMWQ peptide sequences in the figure below. Self-assembled structures for CKAKAQANMWQ are shown in Fig. 10 in the main text. Overall effect of removing N-terminal cysteine on hydrophobicity of the fiber is similar to the results discussed for KIKQIINMWQ.

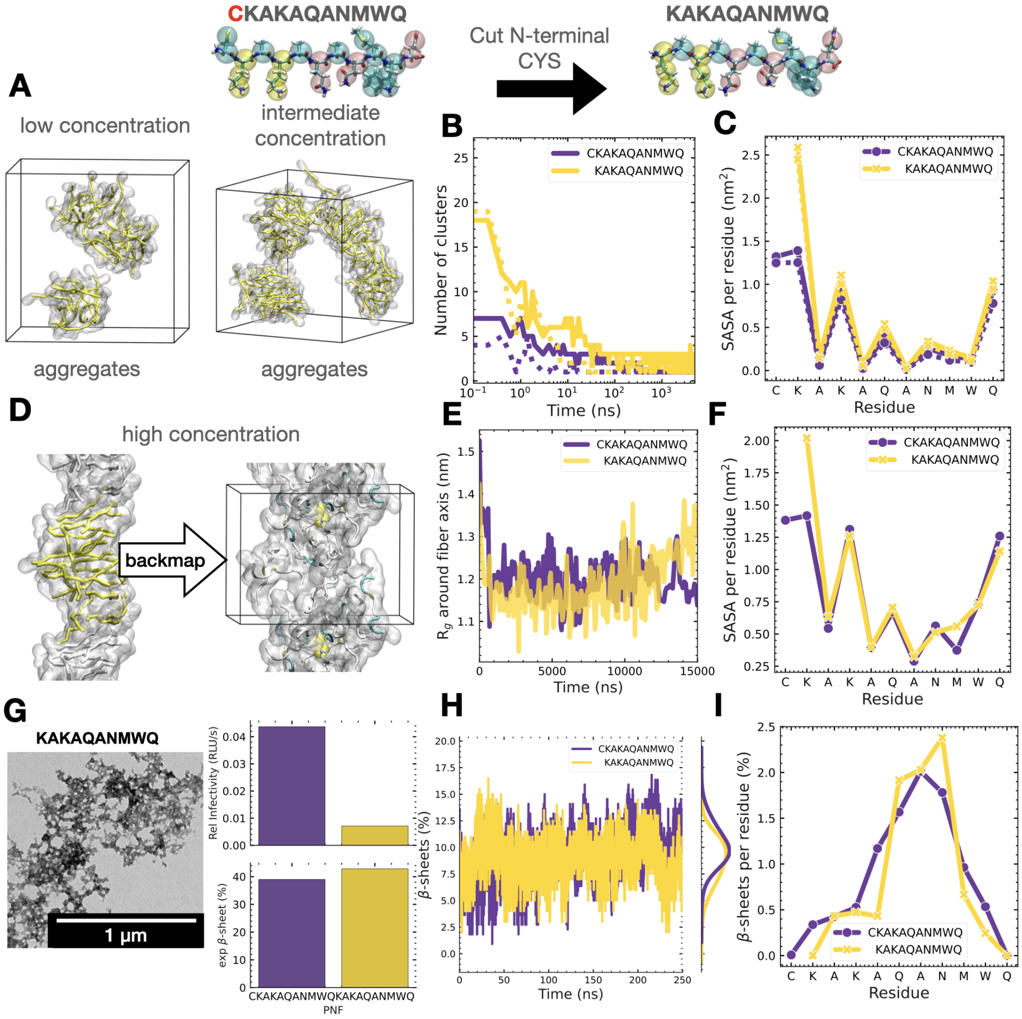

Figure S9: (A) Snapshots from low and intermediate concentration coarse-grained self-assembly simulations after 5 μs for KAKAQANMWQ. Only peptide backbones are colored for easier view of peptide assembly. The side chains are shown in white. Water and ions are omitted for clarity. (B) Number of clusters during self-assembly as a function of simulation time. (C) Average solvent accessible surface area (SASA) per residue calculated from the last 2 μs of simulation. The solid lines refer to simulations at low peptide concentration, while dotted lines refer to simulations at intermediate peptide concentration. (D) Snapshots from after 15 μs of high concentration coarse-grained self-assembly simulation and after 250 ns atomistic simulation after backmapping. (E) The average radius of gyration around the fiber axis from high concentration self-assembly simulation. (F) Average solvent accessible surface area (SASA) per residue calculated from the self-assembled atomistic fiber simulation. (G) TEM images for KAKAQANMWQ aggregates and experimental infecivity relative to EF-C and β-sheet content comparing CKAKAQANMWQ and KAKAQANMWQ. (H) Calculated percentage of β-sheets from self-assembled atomistic fiber simulations. (I) Average percentage of β-sheets per residue.

In the simulations with pre-formed fibers, the results for alanine contaning peptide sequences differ from isoleucine containing peptides, shown in the figure below. Here KAKAQANMWQ peptide shows a high amount of β-sheets structures compared to CKAKAQANMWQ. However, careful investigation of the snapshots from these simulations indicate that KAKAQANMWQ fiber starts to dissociate into small aggregates. Therefore, we expect that these fiber structures for alanine containing peptides is not very stable.

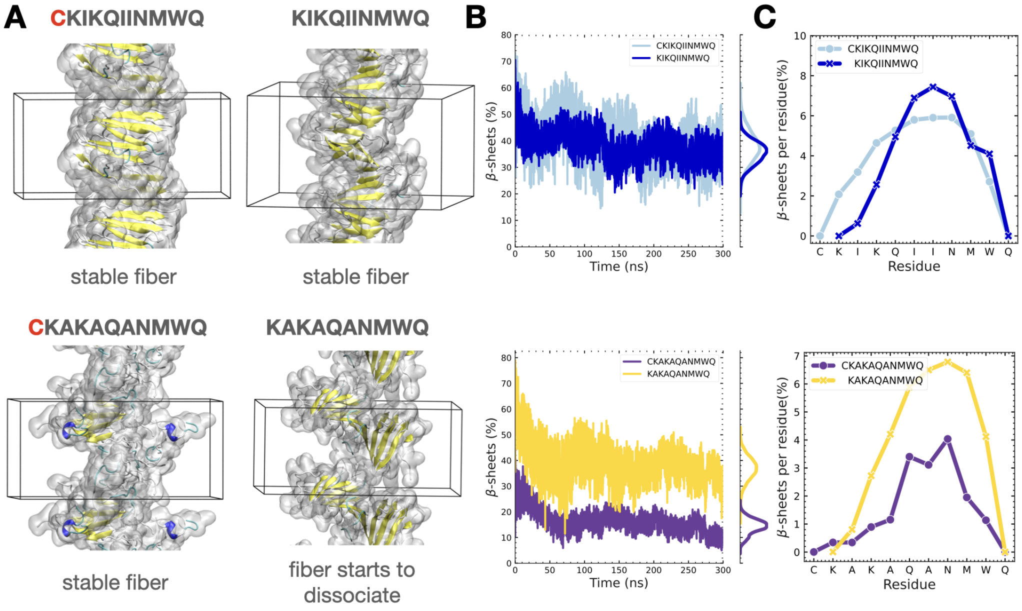

Figure S10: Effect of N-terminal cysteine from pre-formed fiber simulations. (A) Snapshots of side views of CKIKQIINMWQ, KIKQIINMWQ, CKAKAQANMWQ and KAKAQANMWQ pre-formed fibers after 300 ns atomistic simulation. Peptide backbones are drawn and colored according to the secondary structure, where β-sheets are shown in yellow and 3_{10}-helix structures are in dark blue. (B) Total percentage of amino acid residues making β-sheets structures over the simulation and (C) average β-sheet amount per residue calculated from the last 100 ns of simulation. Both in (B) and (C), percentage is calculated with respect to the total number of amino acid residues.

Here, we investigate the effect of switching cysteine from the N-terminus to C-terminus of the peptide. Only when the amphiphilic order is optimized, we observe fiber formation at intermediate and high concentrations for KIKIQIC.

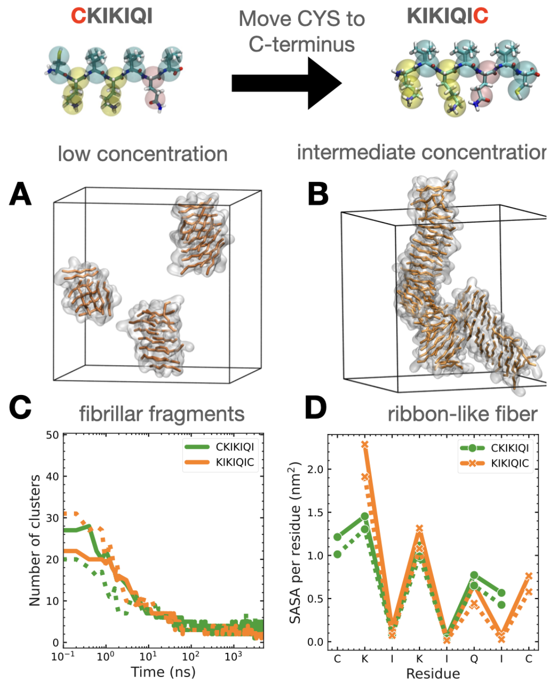

Figure S11: Snapshots from (A) low and (B) intermediate concentration coarse-grained self-assembly simulations after 5 µs for KIKIQIC. Only peptide backbones are colored for easier view of peptide assembly. The side chains are shown in white. Water and ions are omitted for clarity. (C) Number of clusters during self-assembly as a function of simulation time. (D) Average solvent accessible surface area (SASA) per residue calculated from the last 2 µs of simulation. The solid lines refer to simulations at low peptide concentration, while dotted lines refer to simulations at intermediate peptide concentration.

We observed very similar results when we performed simulations for other sequences, such as KIKQIINMWQC and KIKQIIC (Fig. S13-S15). Overall, our results were consistent with KIKIQIC. We did not observe significant differences in properties such as morphology of self-assembled peptides, self-assembly kinetics or amount of β-sheet structures. However, C-terminal cysteine was always more hydrophobic and interacted less with solvent compared with N-terminal cysteine. When we switch the position of the cysteine from N-terminus to C-terminus, the fibrillar morphology of the CKIKQIINMWQ disappears and instead we observe a segment of the fiber that is non-interacting with its periodic image. Only at high concentration, we can form a fiber that remains stable after being backmapped to atomistic representation. The surface hydrophobicity differs at the two termini of the peptide as expected, but the overall β-sheet percentage is similar.

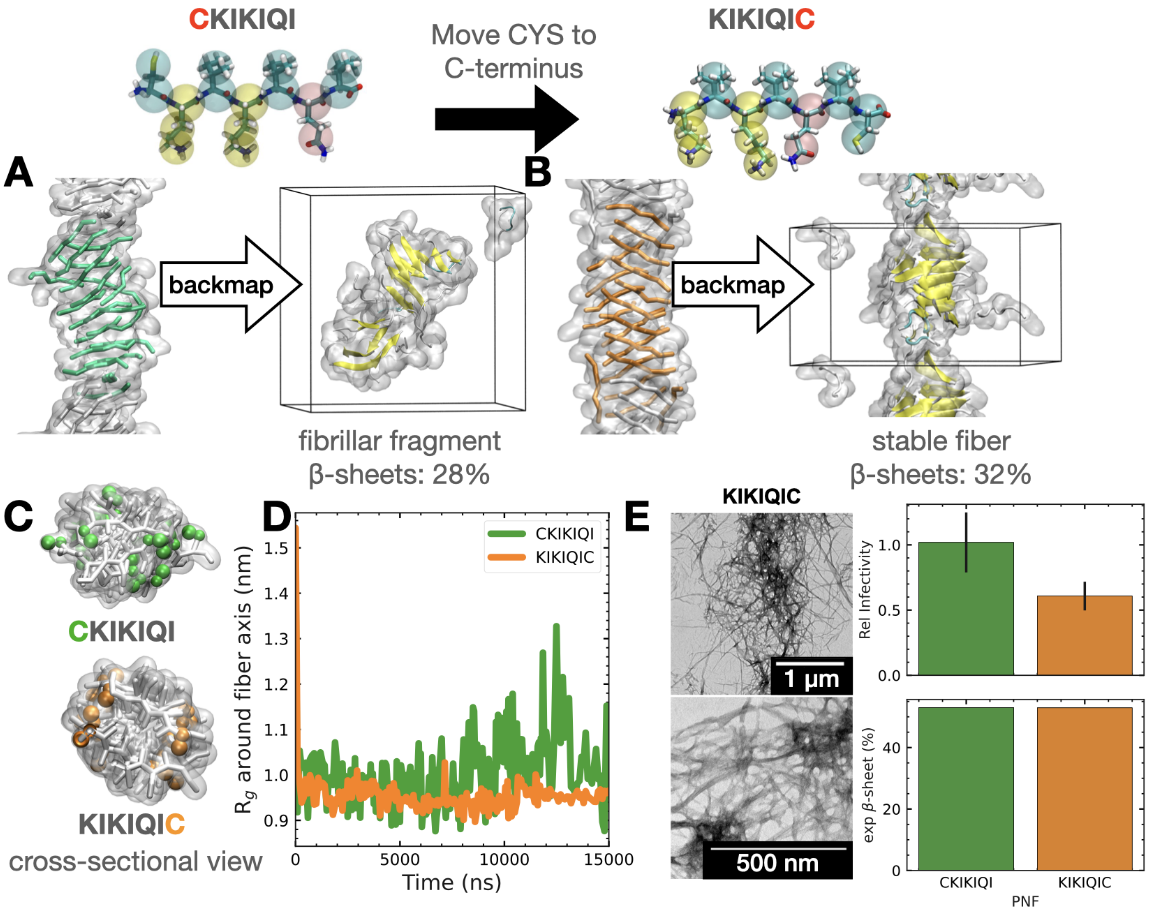

Figure S12: Snapshots of self-assembled fibers in solution from coarse-grained and atomistic simulations for (A) CKIKIQI and (B) KIKIQIC. (C) Cross-sectional view of the self-assembled fibers showing the distribution of cysteine residues. (D) The radius of gyration around the fiber axis from coarse-grained simulations. (E) Experimental TEM images of KIKIQIC, β-sheet content and infectivity relative to EF-C. For TEM images of CKIKIQI, see Fig. S21.

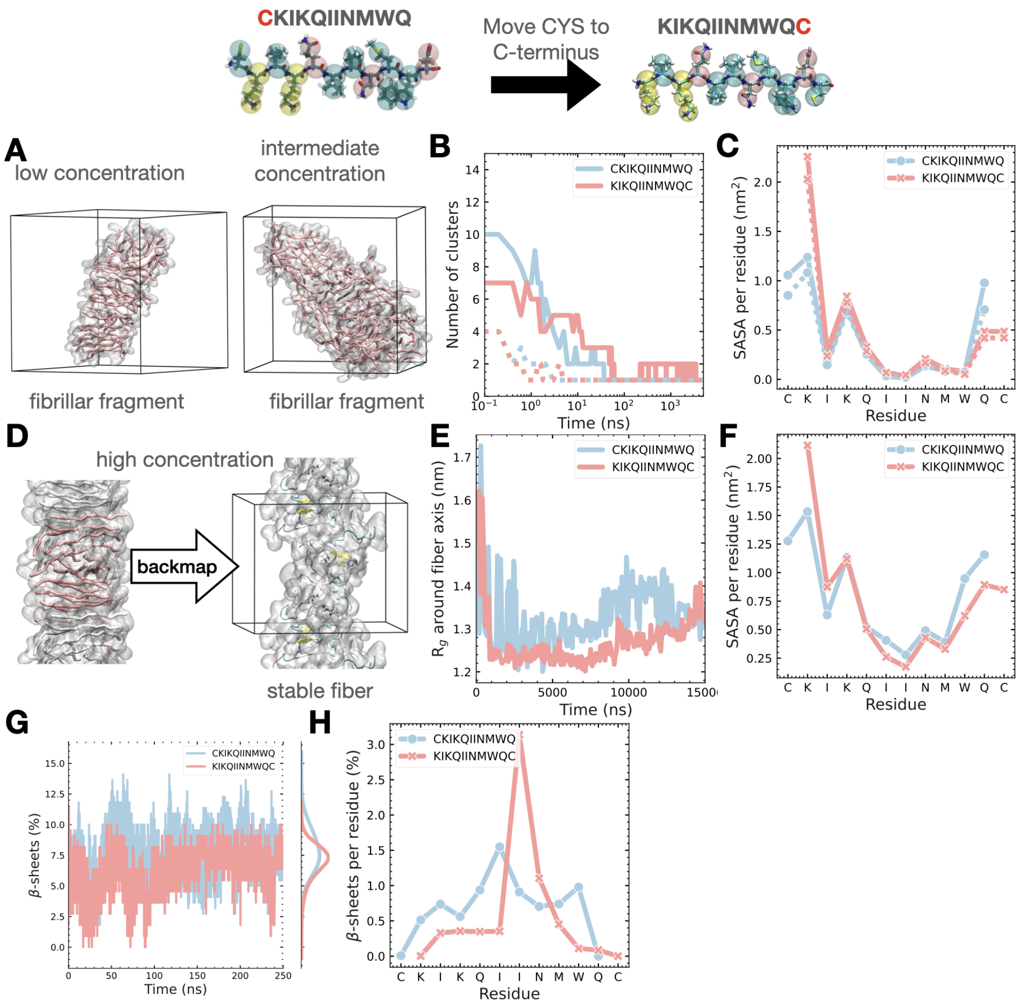

Figure S13: (A) Snapshots from low and intermediate concentration coarse-grained self-assembly simulations after 5 μs for KIKQIINMWQC. Only peptide backbones are colored for easier view of peptide assembly. The side chains are shown in white. Water and ions are omitted for clarity. (B) Number of clusters during self-assembly as a function of simulation time. (C) Average solvent accessible surface area (SASA) per residue calculated from the last 2 μs of simulation. The solid lines refer to simulations at low peptide concentration, while dotted lines refer to simulations at intermediate peptide concentration. (D) Snapshots from after 15 μs of high concentration coarse-grained self-assembly simulation and after 250 ns atomistic simulation after backmapping. (E) The average radius of gyration around the fiber axis from high concentration self-assembly simulation. (F) Average solvent accessible surface area (SASA) per residue calculated from the self-assembled atomistic fiber simulation. (G) Calculated percentage of β-sheets from self-assembled atomistic fiber simulations. (H) Average percentage of β-sheets per residue.

For the short peptide sequences, we don't observe fibrillar structures for any of the tested concentrations. See Fig. S19 and S20 for CKIKQII.

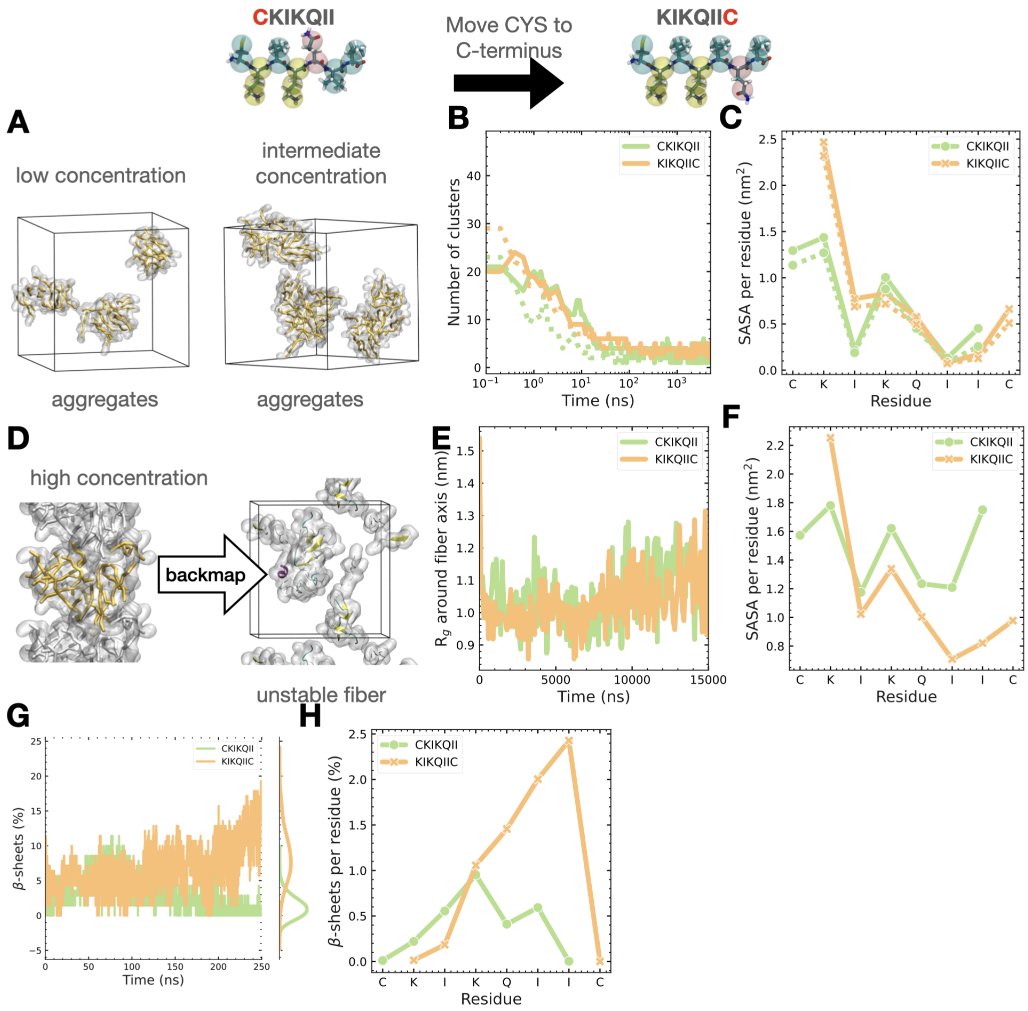

Figure S14: (A) Snapshots from low and intermediate concentration coarse-grained self-assembly simulations after 5 µs for KIKQIIC. Only peptide backbones are colored for easier view of peptide assembly. The side chains are shown in white. Water and ions are omitted for clarity. (B) Number of clusters during self-assembly as a function of simulation time. (C) Average solvent accessible surface area (SASA) per residue calculated from the last 2 µs of simulation. The solid lines refer to simulations at low peptide concentration, while dotted lines refer to simulations at intermediate peptide concentration. (D) Snapshots from after 15 µs of high concentration coarse-grained self-assembly simulation and after 250 ns atomistic simulation after backmapping. (E) The average radius of gyration around the fiber axis from high concentration self-assembly simulation. (F) Average solvent accessible surface area (SASA) per residue calculated from the self-assembled atomistic fiber simulation. (G) Calculated percentage of β-sheets from self-assembled atomistic fiber simulations. (H) Average percentage of β-sheets per residue.

The effect of switching cysteine position is shown below for the pre-formed fiber simulations. Here, we see that the total β-sheet content is similar, but location of β-sheets in the peptide sequence shifts from the N-termius to C-terminus. Thus, once again showing that there is a correlation between surface hydrophobicity and β-sheet formation.

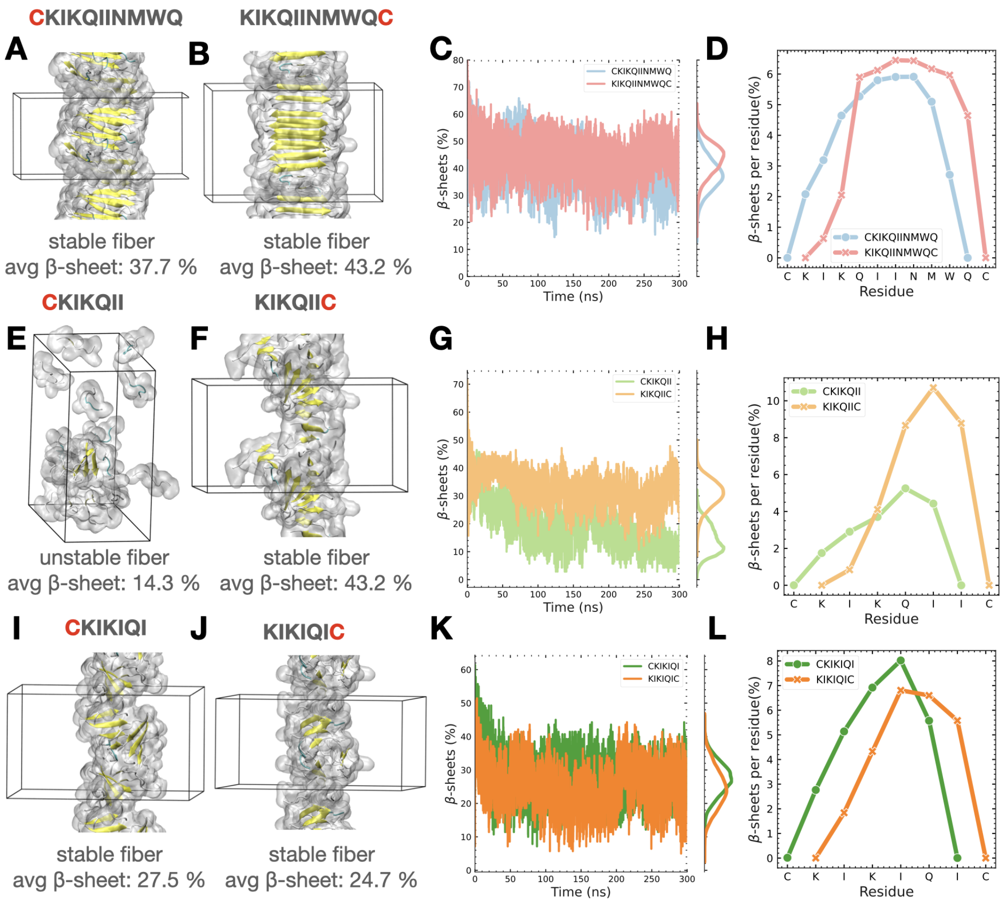

Figure S15: Effect of switching N-terminal cysteine to C-terminus from pre-formed fiber simulations. Snapshots of side views of (A) CKIKQIINMWQ, (B) KIKQIINMWQC, (E) CKIKQII, (F) KIKQIIC, (I) CKIKIQI and (J) KIKIQIC pre-formed fibers after 300 ns atomistic simulation. Peptide backbones are drawn and colored according to the secondary structure, where β-sheets are shown in yellow. (C, G, K) Total percentage of amino acid residues making β-sheets structures over the simulation and (D, H, L) average β-sheet amount per residue calculated from the last 100 ns of simulation. The percentage of β-sheets is calculated with respect to the total number of amino acid residues.

Below, we show the effect of replacing N-terminal cysteine with methionine. Cysteine and methionione are both sulfur containing amino acids, but because methionine has a larger alkyl side chain, it is more hydrophobic. When it comes to our coarse-grained model, both

cysteine and methionine residues are represented with the same bead types, meaning their interaction with the other parts of the peptide and solvent is the same. This is due to the simplification of the coarse-graining procedure. However, since methionine has a bulkier side chain, the bond length between the backbone and side chain bead for methionine is larger compared to the cysteine. As expected, we see no differences between MKIKIQI and CKIKIQI self-assembly in solution for the low and intermediate concentrations, shown below.

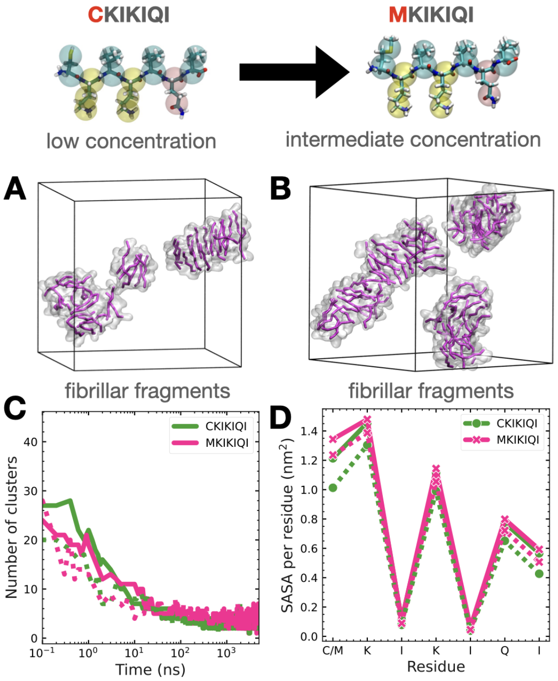

Figure S16: Snapshots from (A) low and (B) intermediate concentration coarse-grained self-assembly simulations after 5 μs for MKIKIQI. Only peptide backbones are colored for easier view of peptide assembly. The side chains are shown in white. Water and ions are omitted for clarity. (C) Number of clusters during self-assembly as a function of simulation time. (D) Average solvent accessible surface area (SASA) per residue calculated from the last 2 μs of simulation. The solid lines refer to simulations at low peptide concentration, while dotted lines refer to simulations at intermediate peptide concentration.

For high concentration and atomistic structure, see Figure S17 below.

If we had better alignment of MKIKIQI peptides, would the increase in hydrophobicity of the methionine with respect to cysteine increase the stability of the fiber in solution? Our pre-formed fiber simulations, where peptides are pre-aligned in solution, reveal that substitution of cysteine with methionine in fact increases the amount of β-sheet structures

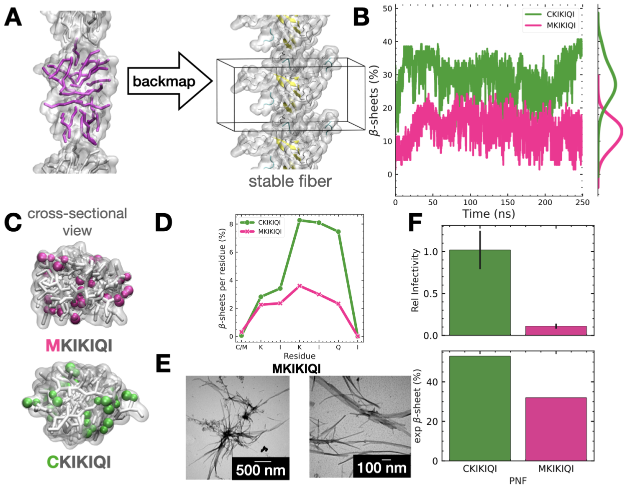

Figure S17: The self-assembled MKIKIQI fiber from coarse-grained and atomistic simulations are shown in (A), where both structures show misalignment of peptides around the periodic boundaries of the simulation box. For comparison to CKIKIQI, see Fig. S12A. The total percentage of β-sheets from are shown in (B). The distribution of the first residue (methionine in pink and cysteine in green) over the cross-section of the fiber is shown in (C). Here, we can see methione residues in the core region disrupting the β-sheet network over the high β-sheet-propensity region (D). Experimental (E) TEM images for MKIKIQI and (F) measured properties.

that form at the core region of the fiber (Fig. S18A-D). This shows that the more hydrophobic methionine amino acid is better at maintaining the stability of the fibrillar morphology by weakening the interaction of the peptides with water and ions, but because of this increased hydrophobicity it can also prefer to be buried near the core of the peptide and lead to misalignment of peptide chains during the self-assembly process, which eventually prevents formation of β-sheet structures. Thus, an intricate balance between hydrophobicity and position of the amino acid in the sequence is needed for optimizing properties of PNFs.

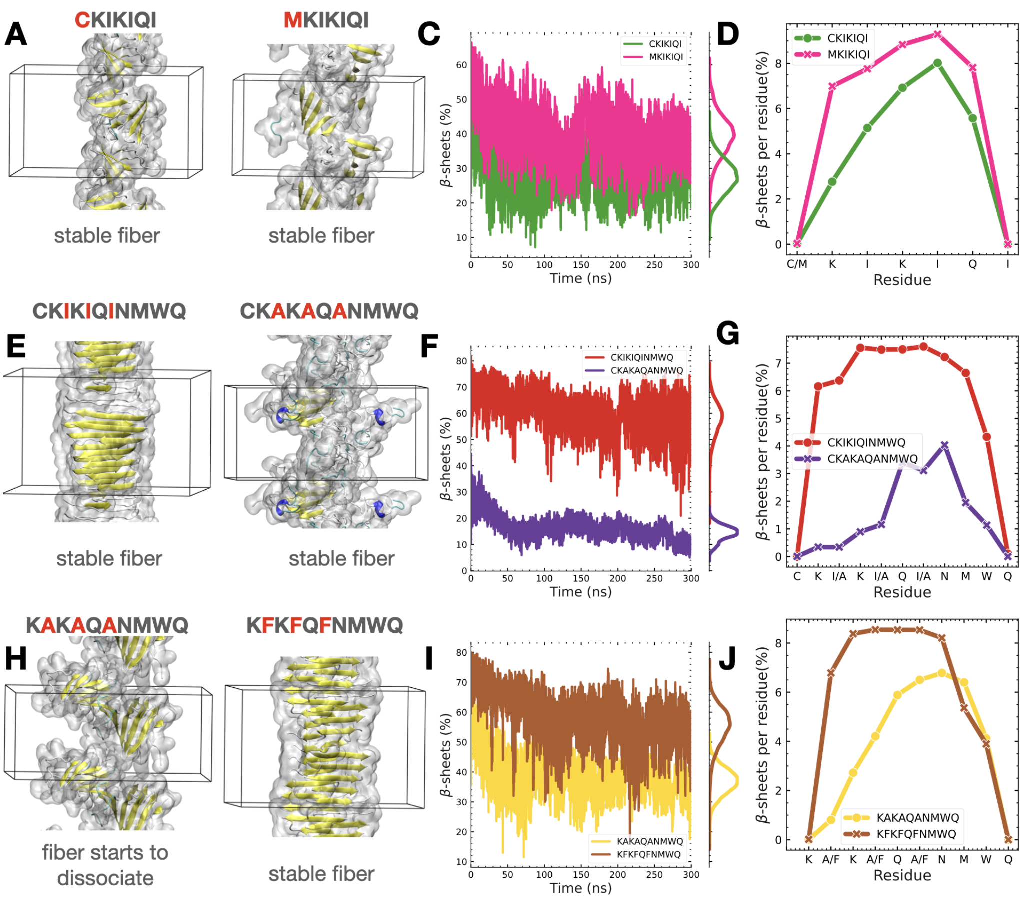

Figure S18: Effect of hydrophobicity from pre-formed fiber simulations. Snapshots of side views of (A) CKIKIQI, MKIKIQI, (E) CKIKIQINMWQ, CKAKAQANMWQ (H) KAKAQANMWQ and KFKFQFNMWQ pre-formed fibers after 300 ns atomistic simulation. Peptide backbones are drawn and colored according to the secondary structure, where β-sheets are shown in yellow and 3_{10}-helix structures are in dark blue. (C, F, I) Total percentage of amino acid residues making β-sheets structures over the simulation and (D, G, J) average β-sheet amount per residue calculated from the last 100 ns of simulation. All β-sheet percentages are calculate with respect to the total number of amino acid residues.

Lack of NMWQ amino acids in the original peptide (CKIKQIINMWQ) results in formation of disordered aggregates at low and intermediate concentrations. The self-assembly of the peptides into the aggregated structures is also slower. After the peptides assemble, the resulting structures have more solvent accessible surface area since they are aggregates in solution instead of a continuous fiber. These aggregates are also visible in the TEM images from experiments.

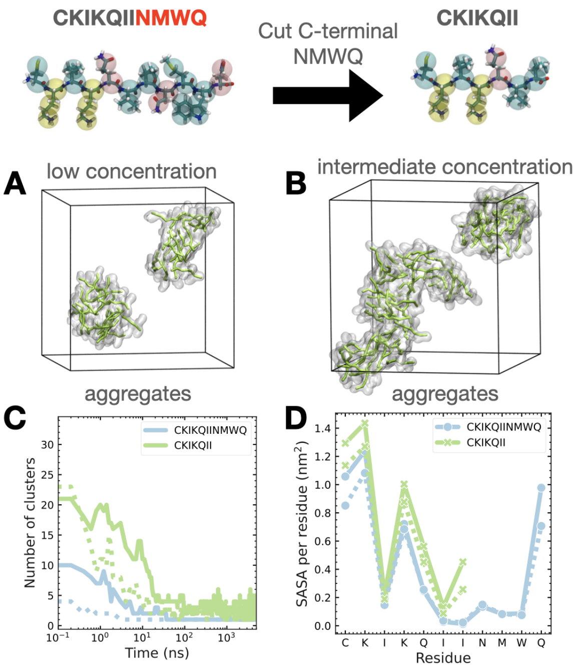

Figure S19: Snapshots from (A) low and (B) intermediate concentration coarse-grained self-assembly simulations after 5 µs for CKIKQII. Only peptide backbones are colored for easier view of peptide assembly. The side chains are shown in white. Water and ions are omitted for clarity. (C) Number of clusters during self-assembly as a function of simulation time. (D) Average solvent accessible surface area (SASA) per residue calculated from the last 2 µs of simulation. The solid lines refer to simulations at low peptide concentration, while dotted lines refer to simulations at intermediate peptide concentration.

At high concentration, the CKIKQII peptides are able to form a fiber, but this structure is not very stable both in coarse-grained system (Movie 2) and atomistic system.

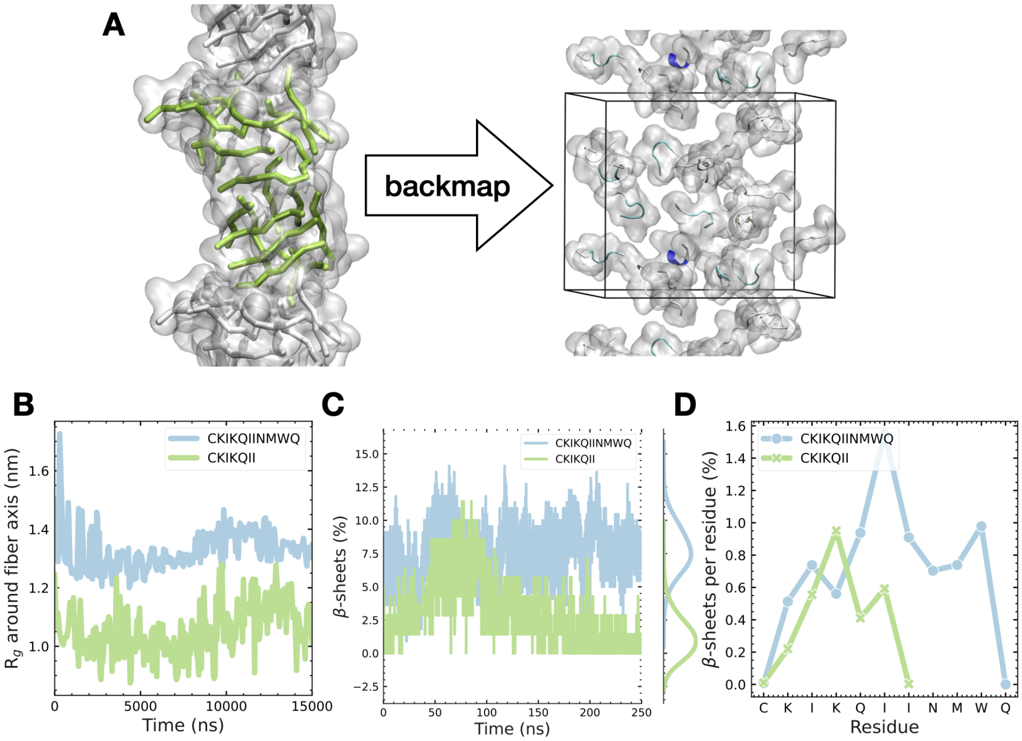

Figure S20: (A) Snapshots from after 15 μs of high concentration coarse-grained self-assembly simulation and after 250 ns atomistic simulation after backmapping for CKIKQII. (B) The average radius of gyration around the fiber axis from high concentration self-assembly simulation. (C) Calculated percentage of β-sheets from self-assembled atomistic fiber simulations. (D) Average percentage of β-sheets per residue.

Even when we start from a pre-formed fiber, CKIKQII loses stability and β-sheet structures very rapidly within about 100 ns, shown in Figure S21A. Nevertheless, this does not mean that all short peptide sequences perform poorly. A single change in the order of amino acids, i.e. CKIKQII to CKIKIQI, enhances the stability and β-sheet propensity of peptides. There is a decrease in amount of β-sheets when NMWQ is removed (Fig. S21B), but CKIKIQI still has a fibrillar morphology with comparable β-sheet composition to the original sequence CKIKQIINMWQ. In experiments, there is a decrease in infectivity and β-sheet formation for CKIKQIINMWQ to CKIKQII transformation, which is not true for CKIKIQI (Fig. S21C-D). TEM images also show considerable differences in the morphologies of CKIKIQI, where thicker, more-defined fibers are present. The effect of amphiphilic pattern in PNF structure is discussed in the next section in more detail.

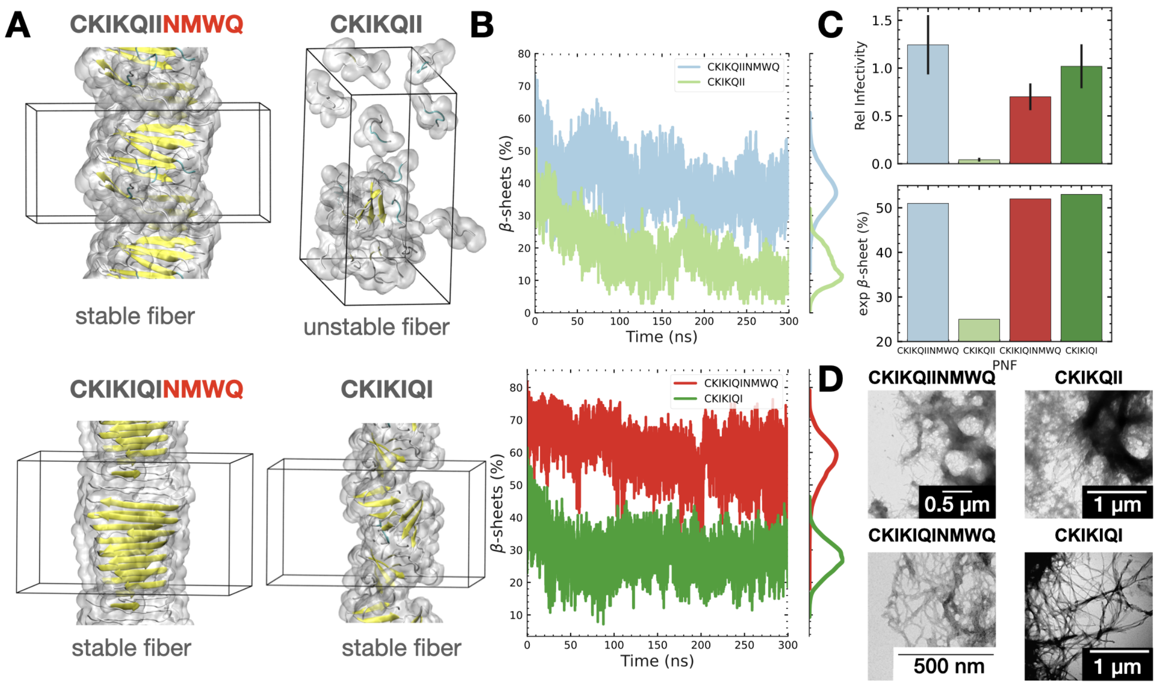

Figure S21: (A) Snapshots of pre-formed fibers after 300 ns of atomistic simulations and (B) the corresponding percentage of total β-sheets, showing the effect of NMWQ on structure formation. Experimental data on (C) infectivity relative to EF-C and β-sheet content and (D) TEM images for the chosen peptide sequences.

Fibril core hydrophobicity increases order and β-sheet formation

Changing the internal order of the amino acids from CKIKQII to CKIKIQI results small segments that are Amyloid-like in the way they are aligned, instead of disordered aggregates shown in Fig. S19. A fiber forms at high concentration, which loses its periodicity after 250 ns of atomistic simulation (Fig. S12A). However, this small segment of fiber still retains a high percentage of β-sheets compared to other peptide sequences. These β-sheets correspond to the core region of the peptide sequence where the intrinsic switch takes place. Coarse-grained simulations at low and intermediate concentrations did not show a significant difference in self-assembly kinetics or the solvent accessible surface area, but the peptides that form the aggregates in solution were more ordered in an amyloid-like packing for CKIKIQI (Fig. S22). TEM images also show considerable differences in the morphologies of CKIKIQI, where thicker, more-defined fibers are present.

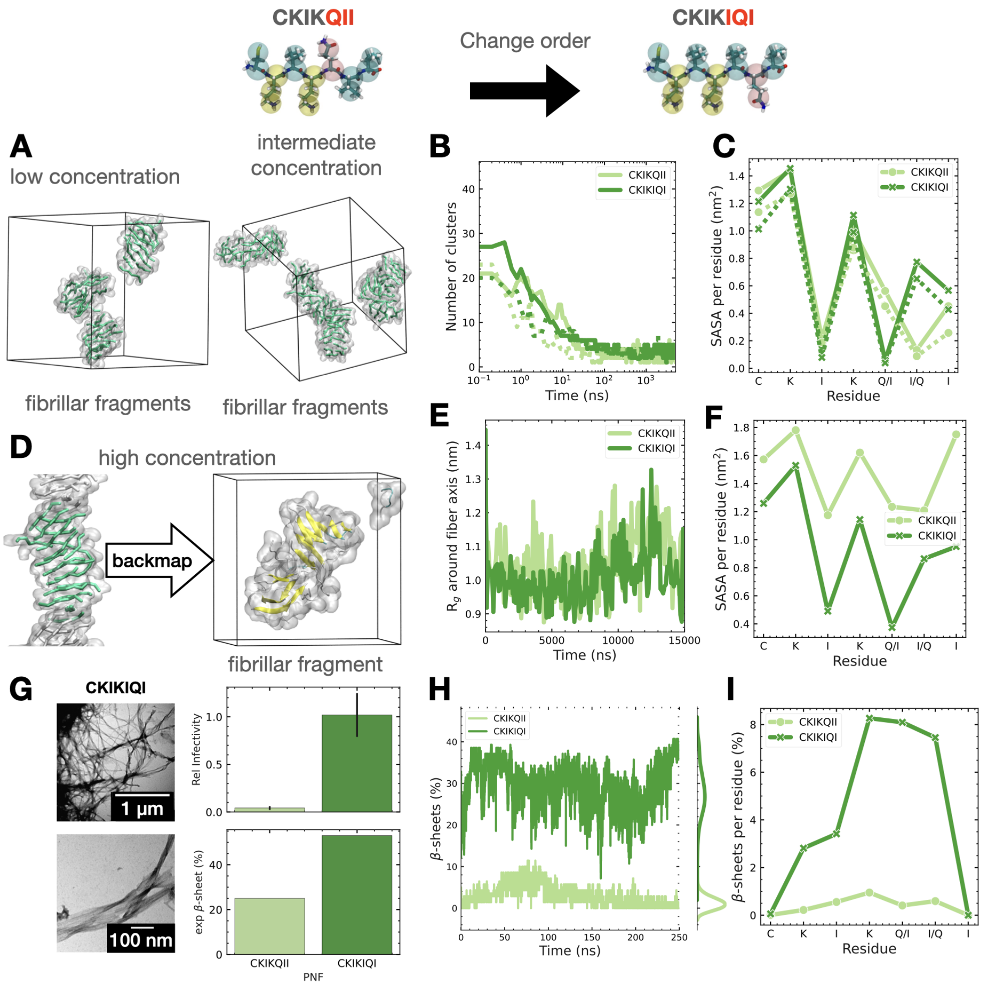

Figure S22: (A) Snapshots from low and intermediate concentration coarse-grained self-assembly simulations after 5 μs for CKIKIQI. Only peptide backbones are colored for easier view of peptide assembly. The side chains are shown in white. Water and ions are omitted for clarity. (B) Number of clusters during self-assembly as a function of simulation time. (C) Average solvent accessible surface area (SASA) per residue calculated from the last 2 μs of simulation. The solid lines refer to simulations at low peptide concentration, while dotted lines refer to simulations at intermediate peptide concentration. (D) Snapshots from after 15 μs of high concentration coarse-grained self-assembly simulation and after 250 ns atomistic simulation after backmapping. (E) The average radius of gyration around the fiber axis from high concentration self-assembly simulation. (F) Average solvent accessible surface area (SASA) per residue calculated from the self-assembled atomistic fiber simulation. (G) TEM images for CKIKIQI aggregates and experimental infecivity relative to EF-C and β-sheet content comparing CKIKQII and CKIKIQI. (H) Calculated percentage of β-sheets from self-assembled atomistic fiber simulations. (I) Average percentage of β-sheets per residue.

For the original sequence, optimizing the amphiphilic pattern has the effect shown below. Overall morphology and surface hydrophobicity is similar. The self-assembly kinetics for the

low and intermediate concentrations is between those of CKIKQIINMWQ. In experiments, we did not observe a significant difference in the infection rates.

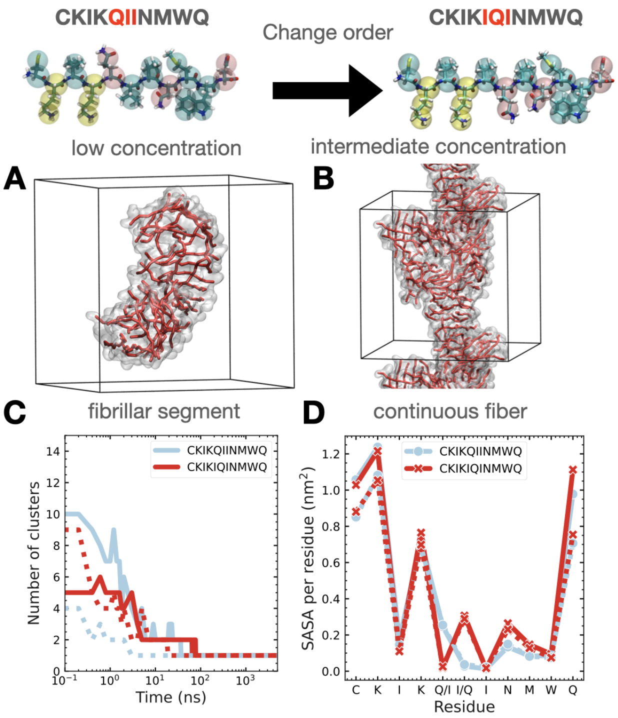

Figure S23: Snapshots from (A) low and (B) intermediate concentration coarse-grained self-assembly simulations after 5 μs for CKIKIQINMWQ. Only peptide backbones are colored for easier view of peptide assembly. The side chains are shown in white. Water and ions are omitted for clarity. (C) Number of clusters during self-assembly as a function of simulation time. (D) Average solvent accessible surface area (SASA) per residue calculated from the last 2 μs of simulation. The solid lines refer to simulations at low peptide concentration, while dotted lines refer to simulations at intermediate peptide concentration.

At high concentration, we observe a slight increase in the total number of β-sheets. This change corresponds to the core region of the peptide sequence, where the number of β-sheets increases continuously up to residue 5, which is where the intrinsic switch happens, plateaus and then starts to decrease after residue 7 toward the C-terminus of the peptide chain.

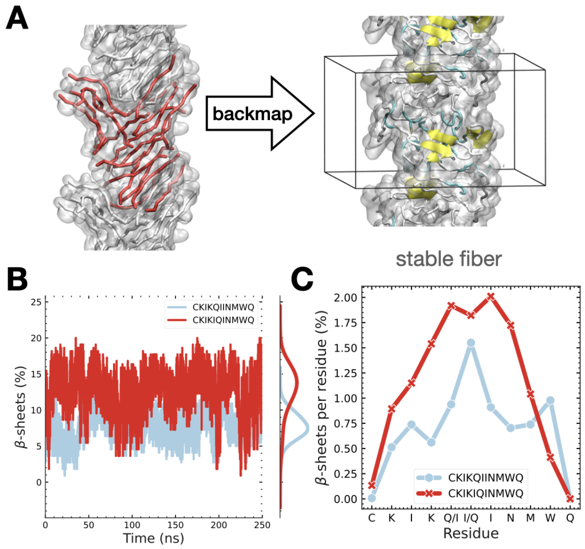

Figure S24: (A) Snapshots from after 15 μs of high concentration coarse-grained self-assembly simulation and after 250 ns atomistic simulation after backmapping for CKIKIQINMWQ. (B) The average radius of gyration around the fiber axis from high concentration self-assembly simulation. (C) Calculated percentage of β-sheets from self-assembled atomistic fiber simulations. (D) Average percentage of β-sheets per residue.

We also replaced the isoleucine in CKIKIQINMWQ peptide with alanine, which is less hydrophobic and has a smaller side chain. Our simulations revealed that CKAKAQANMWQ peptides assembled into aggregates with peptides not as ordered as in CKIKIQINMWQ aggregates, shown in Figure S25. CKIKIQINMWQ structures resemble a fibrillar fragment at low concentration and a twisted fibril at intermediate concentration, where the peptides are stacked mostly perpendicular to the long axis of the fiber. CKAKAQANMWQ, on the other hand, self-assembles to form aggregates that are not continuous over the periodic box in any direction, and the peptide is more randomly packed together. Interestingly, the self-assembly kinetics calculated by number of clusters over time are very similar for both peptides, but the polar residues close to the core of the fiber (i.e K and Q) have slightly more surface area accessible to the solvent when they are found around alanine instead of isoleucine (Fig. S25C-D). This small decrease in hydrophobicity near the core region of the peptide when isoleucine is replaced by alanine results in more peptide-water interactions that may lead to the less ordered aggregated structures (Fig. S25E).

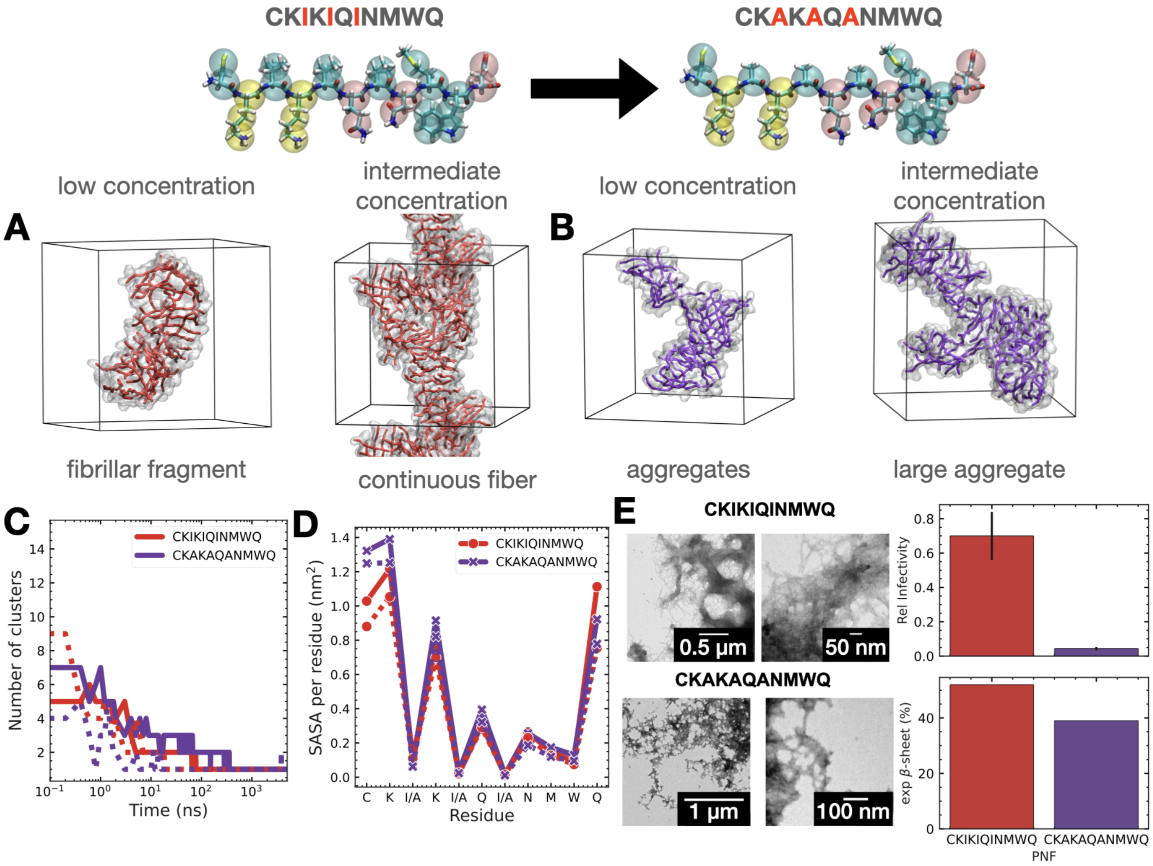

Figure S25: Comparison of self-assembled structures from low and intermediate concentrations of (A) CKIKIQINMWQ and (B) CKAKAQANMWQ peptides. Although the (C) self-assembly kinetics and (D) solvent accessible surface area (SASA) are very similar for both sequences, the morphology of the resulting structures in (A-B) simulations and (E) experiments, as well as experimental β-sheet content and infectivity relative to EF-C are different.

The self-assembled single fiber at high peptide concentration is shown below. Here, we can see that after backmapping to atomistic model, there is a loss of stability in CKAKAQANMWQ, as well as β-sheet formation. For the effect of replacing alanine with more hydrophobic phenylalanine, see the main text.

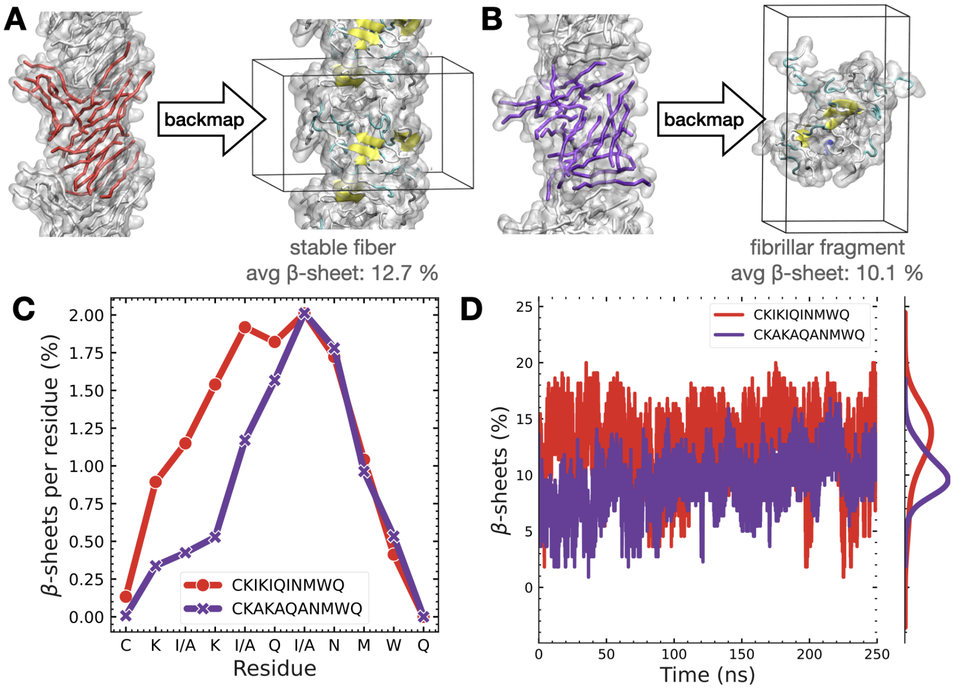

Figure S26: Snapshots from after 15 μs of high concentration coarse-grained self-assembly simulation and after 250 ns atomistic simulation after backmapping for (A) CKIKIQINMWQ and (B) CKAKAQANMWQ. (C) Average percentage of β-sheets per residue. (D) Calculated percentage of β-sheets from self-assembled atomistic fiber simulations.

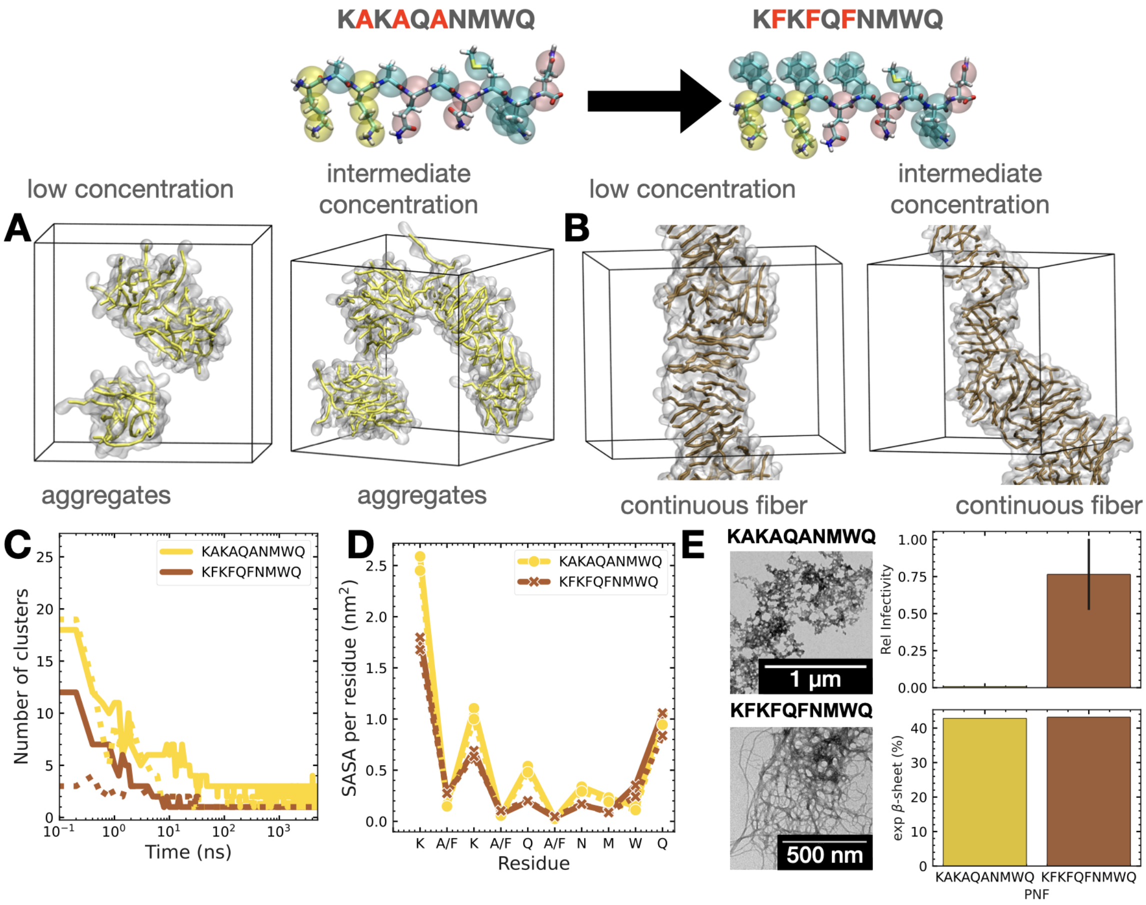

Figure S27: Comparison of self-assembled structures from low and intermediate concentrations of (A)KAKAQANMWQ and (B) KFKFQFNMWQ peptides. There are significant differences in (C) self-assembly kinetics and (D) solvent accessible surface area (SASA), where the increase in hydrophobicity in phenylalanine hinders peptide-water interactions of hydrophilic residues (K and Q) in close proximity. (E) The TEM images and experimentally measured properties show significant enhancement of properties with KFKFQFNMWQ.

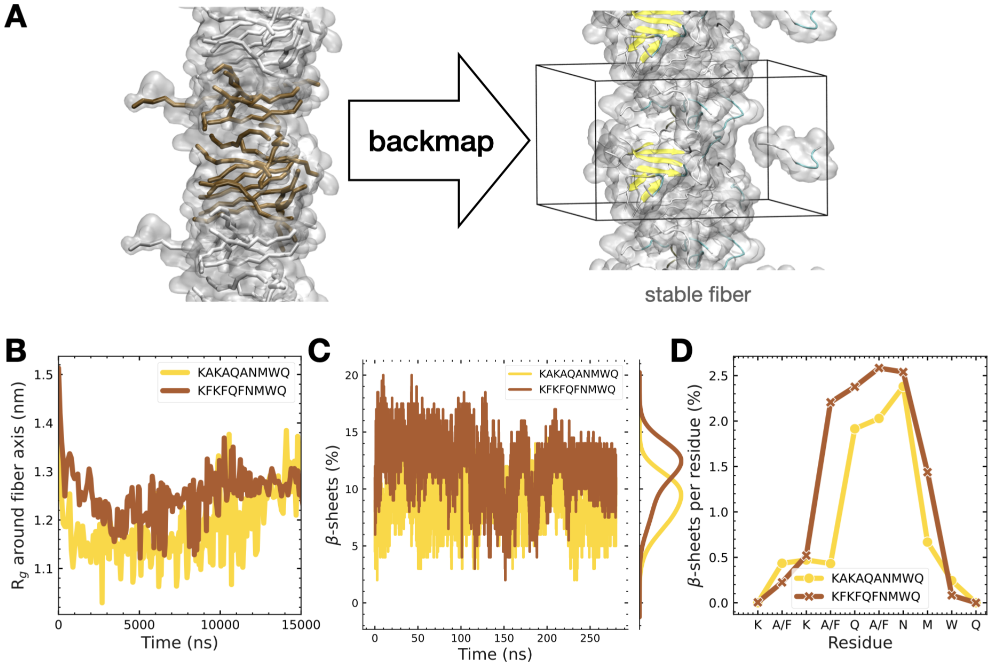

Figure S28: (A) Snapshots from after 15 µs of high concentration coarse-grained self-assembly simulation and after 250 ns atomistic simulation after backmapping for KFKFQFNMWQ. (B) The average radius of gyration around the fiber axis from high concentration self-assembly simulation. (C) Calculated percentage of β-sheets from self-assembled atomistic fiber simulations. (D) Average percentage of β-sheets per residue.

Fibril-fibril interactions promote hierarchical PNF assembly and bioactivity

In order to see whether fibers in solution interact with one another and form larger structures in solution, we placed four fibers in close proximity from each other. The cross-sectional view of the initial structure for CKIKQIINMWQ is shown in Figure S1. For each sequence, we used the coordinates of a stable, self-assembled single fiber from high concentration coarse-grained simulations as initial configurations.

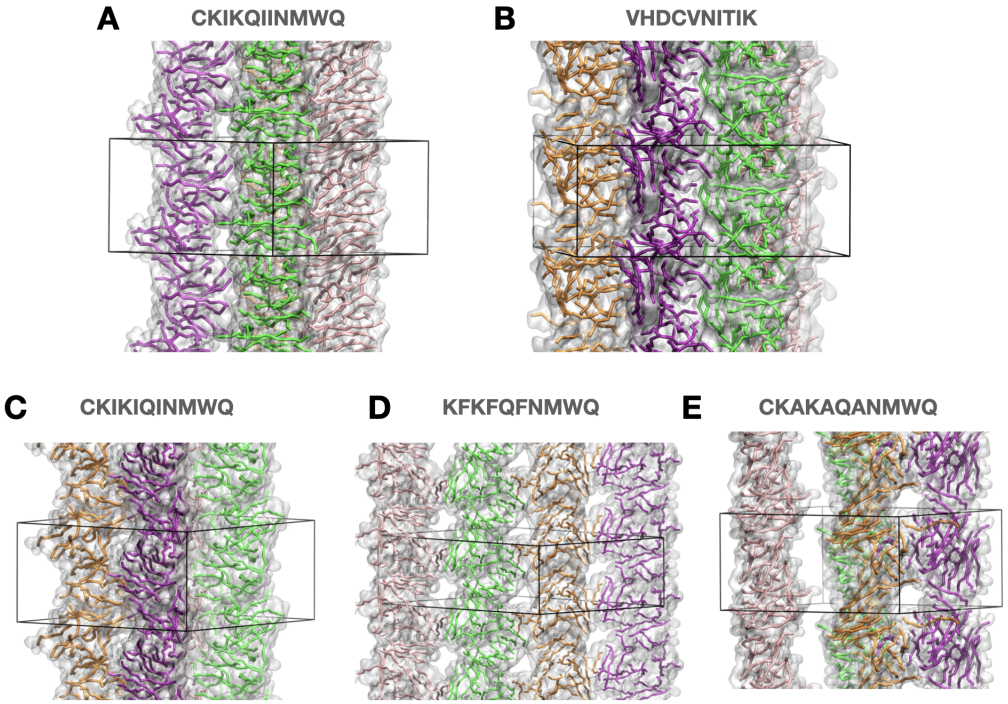

Figure S29: Snapshots of the side view of (A) CKIKQIINMWQ, (B) VHDCVNITIK, (C) CKIKIQINMWQ, (D) KFKFQFNMWQ and (E) CKAKAQANMWQ after 10 µs of coarse-grained multi-fiber simulations. Peptides that belong to the same fiber at time = 0 have same color backbones. The side chains of peptides are shown in white. The simulation box boundaries are drawn in black. Water and ions are omitted for clarity.

Simulations performed by Sasselli et al.[S9] suggested that inter-fiber interactions are enhanced by presence of hydrophilic groups on surfaces and are weakened when they are replaced by hydrophobic ones. However, our simulations show otherwise where removing N-terminal cysteine creates a more hydrophilic nanofibril surface, which prevents aggrega-

tion of peptides in solution (Fig. S30). Furthermore, switching the position of N-terminal cysteine to the C-terminus of the peptide increases intra-fiber cysteine-lysine interactions, neutralizing the surface charge. This in turn results in weakening of inter-fiber interactions and increases the distance between fibers in solution (Fig. S32,S31).

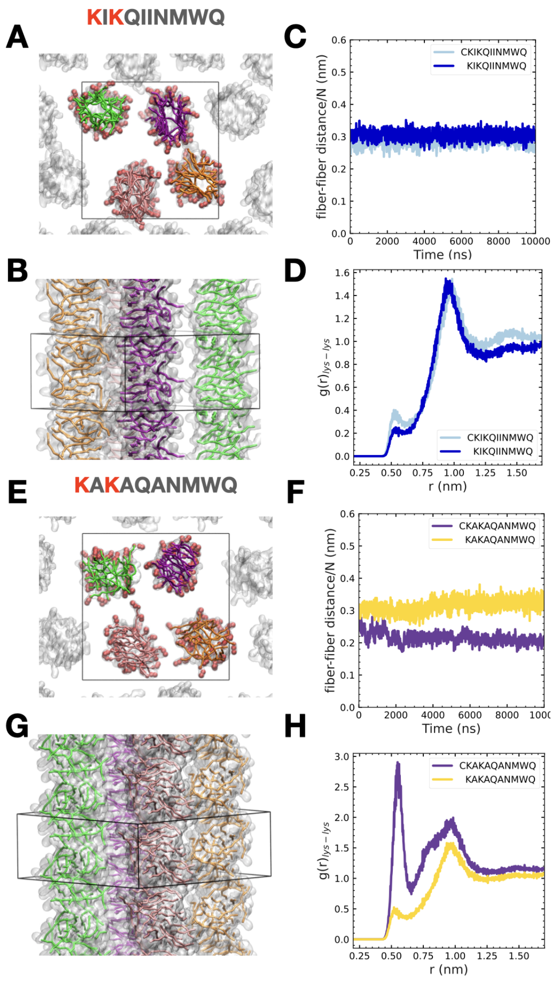

Figure S30: Effect of removing N-terminal cysteine on fiber-fiber interactions. The (A,E) cross-sectional and (B,G) side views of KIKQIINMWQ and KAKAQANMWQ fibers, respectively, in solution. Peptides that belong to the same fiber at time = 0 have same color backbones. The side chains of peptides are shown in white. The simulation box boundaries are drawn in black. Water and ions are omitted for clarity. (C, F) The fiber-fiber distance between the initial structures over the simulation time. (D, H) The intermolecular pair distribution functions, g(r), between lysine groups.

We previously discussed how the lack of NMWQ sequence in the short peptides can result in loss of stability in solution. This is also true in presence of other fibers in solution, shown

in Figure S31, where for both CKIKQII and CKIKIQI, the peptides in different fibers can interact and exchange to form aggregated structures. We should note that even though the initial fiber structures lose their stability, peptides from different fibers interact to form new stable fibers in solution. If we examine the newly formed fibers more closely, we see that CKIKQII forms three structures that are of different sizes, isolated in solution (Fig. S31A,D). The CKIKQII fibers do not aggregate further in the course of 10 μs of simulation. On the other hand, CKIKIQI re-assembles to form an interacting network of fibers in solution, where the supramolecular structure resembles a cross-linked network of fibers rather than isolated fibers in solution (Fig. S31B,E). Furthermore, by moving the N-terminal cysteine to C-terminus, the loss of stability in CKIKIQI is not observed in KIKIQIC, shown in Figure S31C,F. Two of the KIKIQIC fibers aggregate to form a single long fiber, while the rest remain stable and isolated in solution. The aggregation of the fibers in solution can be quantified by the number of clusters that are present (Fig. S31G), where we use a cutoff distance of 0.5 nm for peptides to be considered in the same cluster according to first peak in g(r) in Fig. S31H. The tendency to aggregate is highest for CKIKIQI compared to other sequences, which is also observed in light scattering measurements that indicate the highest microscopic aggregation for CKIKIQI (Fig. S31I).

Although both CKIKQII and KIKIQIC aggregate into three isolated fibers in solution, their morphologies are very different. CKIKQII fibers differ in size with less ordered peptides, while KIKIQIC fibers are all similar and exhibit amyloid-like arrangement with peptides stacked perpendicularly with respect to the length of the fiber. KIKIQIC fibers owe their stability to the perfectly aligned peptides where the N-terminal lysine (+ charge) interact directly with C-terminal cysteine (- charge), visible in the very large magnitude of lysine-cysteine peak in Figure S31H. However, there is very little surface to surface inter-fiber interaction, which is also observed in experiments (Fig. S31I). Interestingly, although KIKIQIC has a high β-sheet content and is highly stable, its infection rates are not as high as CKIKIQI in experiments. Our simulations confirm our previous experimental observation

that microscopic aggregation is another factor that is important for enhancing efficiency of PNFs in retroviral transfer.[S10] Therefore, not only β-sheet formation but also fiber-fiber interactions in PNFs may be a strong indication of their ability to interact with cell membranes and thus improve biological activity.

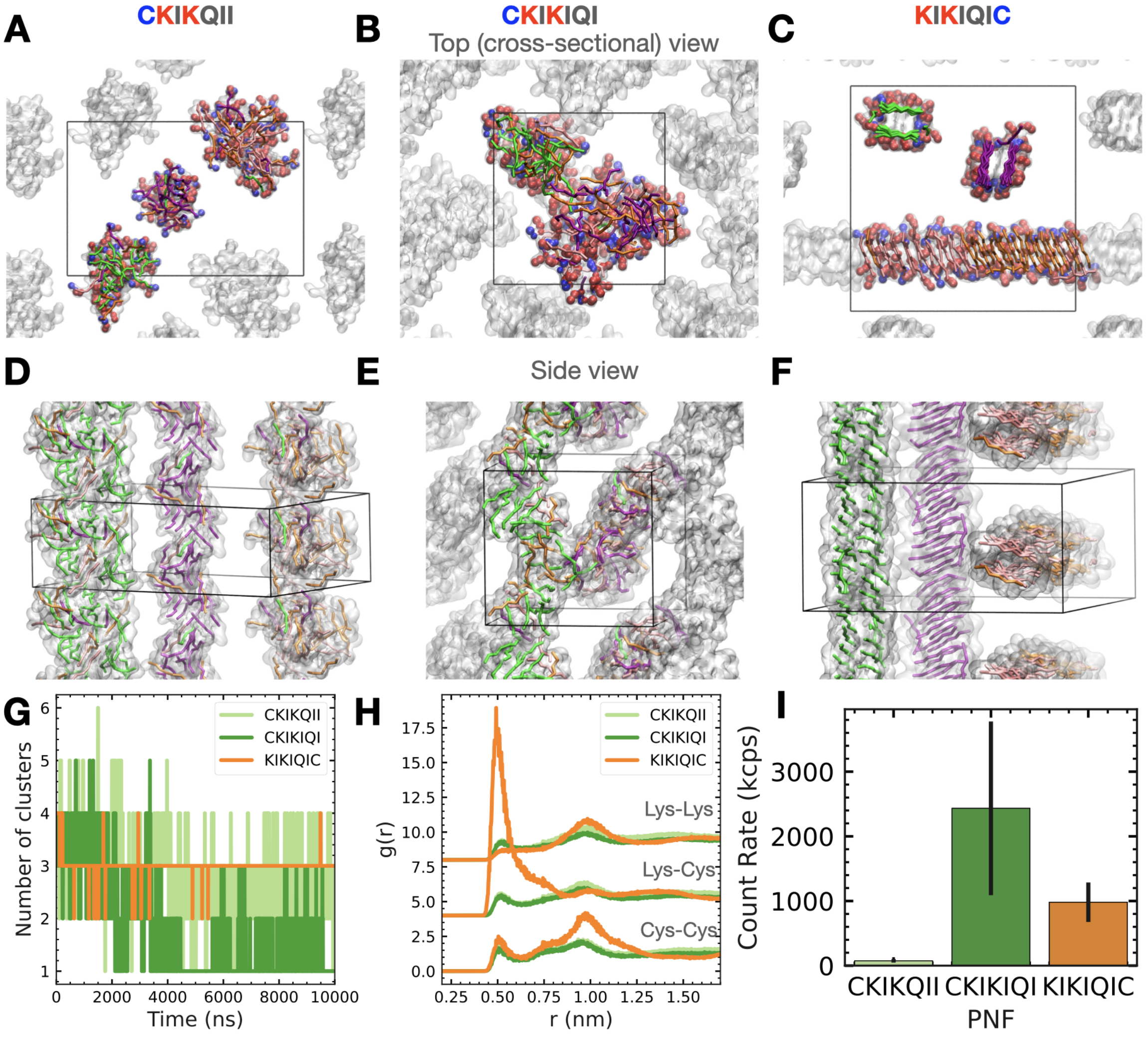

Figure S31: The (A, B, C) cross-sectional and (D, E, F) side views of CKIKQII, CKIKIQI and KIKIQIC fibers, respectively, in solution. The peptide backbones that belong to the same fiber in the initial structure are colored with the same color. Cysteine residues are colored in blue, lysine residues are red. Size of the simulation box is shown in black to show the interaction over periodic images. The side chain beads of the peptide are colored in white. Water and ions are omitted for clarity. (G) The number of clusters (i.e. fibers) over the simulation time, showing remixing and formation of new fibrillar structures. (H) The intermolecular pair distribution functions, g(r), between lysine and cysteine groups. (I) Count rate of scattered light from zeta-potential measurements, indicating microscopic aggregation.[S10]

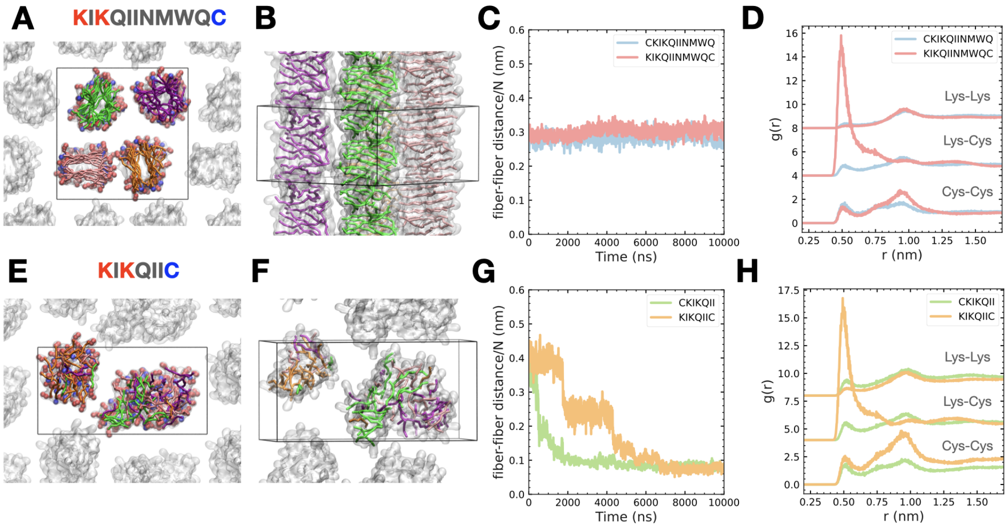

Figure S32: Effect of switching N-terminal cysteine to C-terminus on fiber-fiber interactions. The (A,E) cross-sectional and (B,F) side views of KIKQIINMWQC and KIKQIIC fibers, respectively, in solution. Peptides that belong to the same fiber at time = 0 have same color backbones. The side chains of peptides are shown in white. The simulation box boundaries are drawn in black. Water and ions are omitted for clarity. (C, G) The fiber-fiber distance between the initial structures over the simulation time. (D, H) The intermolecular pair distribution functions, g(r), between lysine and cysteine groups.

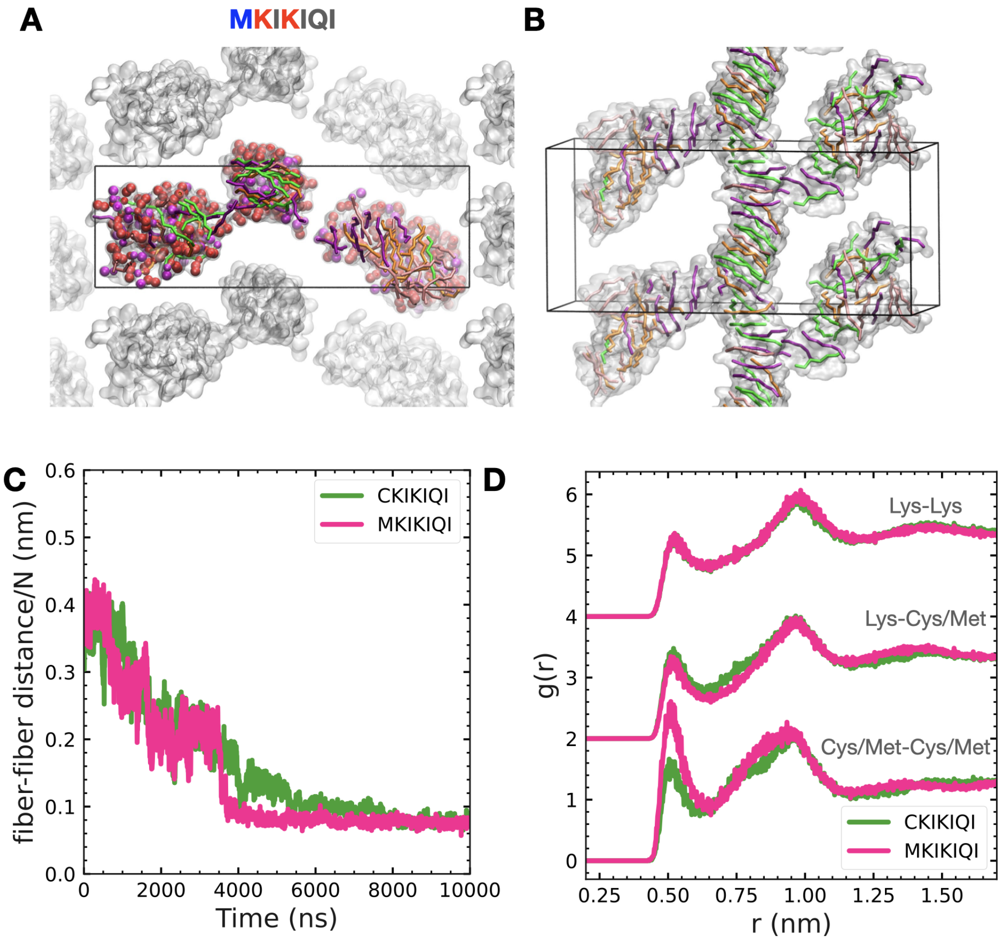

Figure S33: Effect of cysteine to methionine mutation on fiber-fiber interactions. The (A) cross-sectional and (B) side views of MKIKIQI fibers, respectively, in solution. Peptides that belong to the same fiber at time = 0 have same color backbones. The side chains of peptides are shown in white. The simulation box boundaries are drawn in black. Water and ions are omitted for clarity. (C) The fiber-fiber distance between the initial structures over the simulation time. (D) The intermolecular pair distribution functions, g(r), between lysine and cysteine/methionine groups.

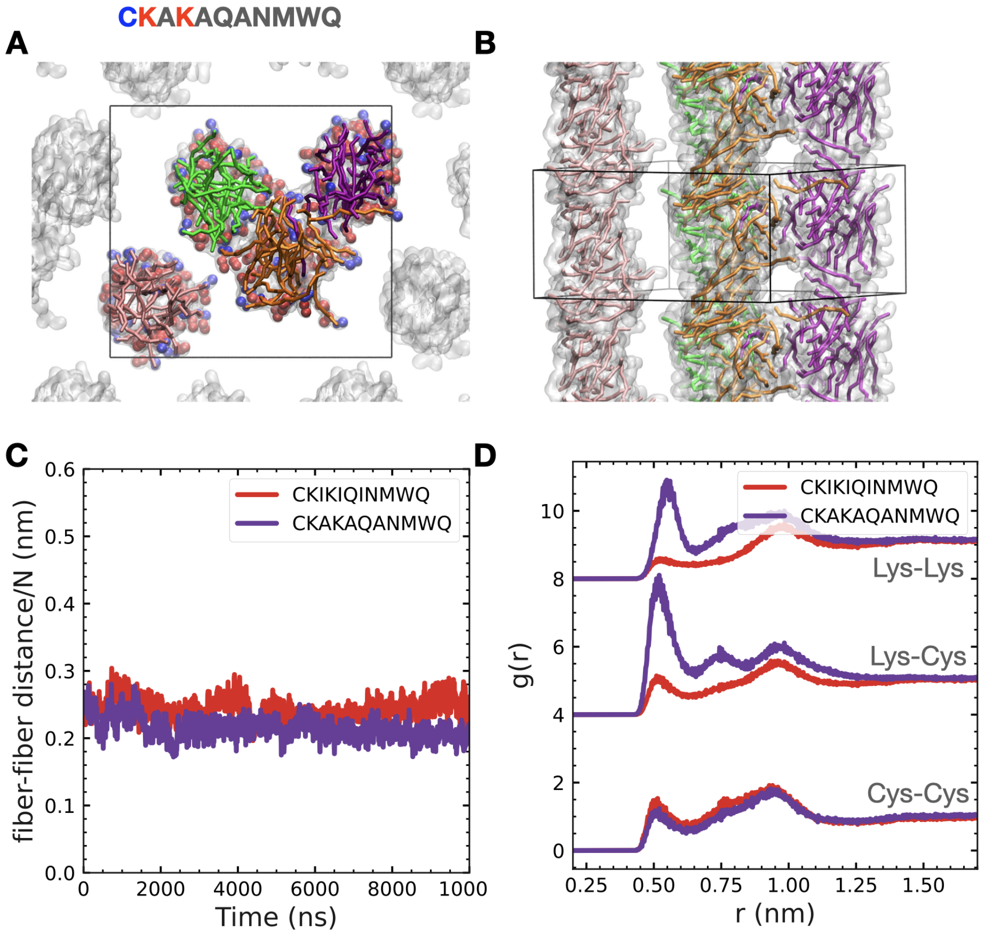

Figure S34: Effect of isoleucine to alanine mutation on fiber-fiber interactions. The (A) cross-sectional and (B) side views of CKAKAQANMWQ fibers, respectively, in solution. Peptides that belong to the same fiber at time = 0 have same color backbones. The side chains of peptides are shown in white. The simulation box boundaries are drawn in black. Water and ions are omitted for clarity. (C) The fiber-fiber distance between the initial structures over the simulation time. (D) The intermolecular pair distribution functions, g(r), between lysine and cysteine groups.

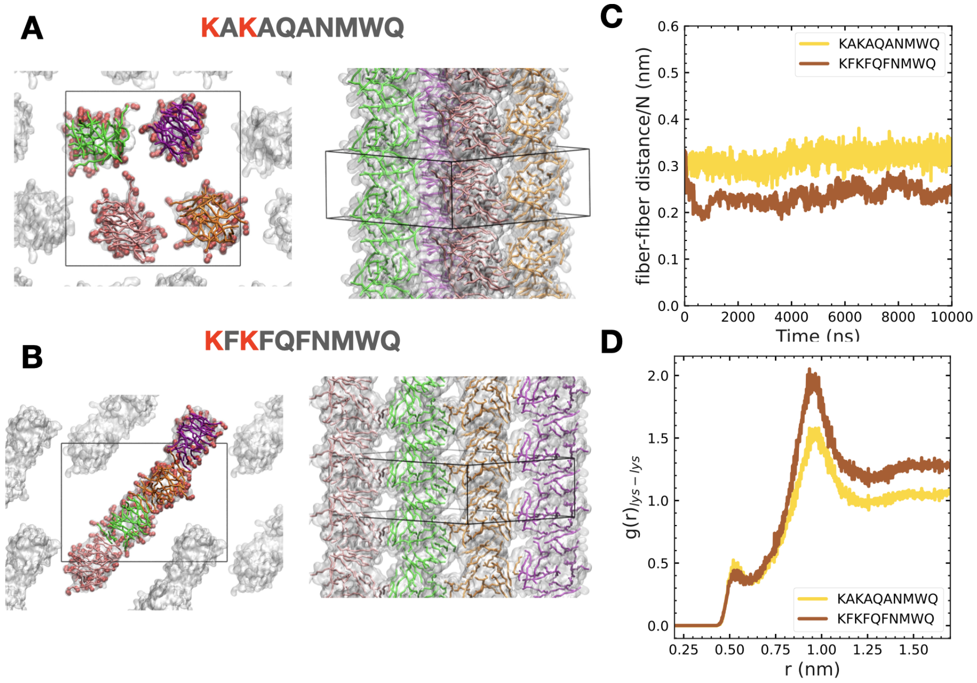

Figure S35: Effect of alanine to phenylalanine mutation on fiber-fiber interactions. The cross-sectional and side views of (A) KAKAQANMWQ and (B) KFKFQFNMWQ fibers, respectively, in solution. Peptides that belong to the same fiber at time = 0 have same color backbones. The side chains of peptides are shown in white. The simulation box boundaries are drawn in black. Water and ions are omitted for clarity. (C) The fiber-fiber distance between the initial structures over the simulation time. (D) The intermolecular pair distribution functions, g(r), between lysine groups.

As briefly discussed in the section "β-sheet formation in self-assembly of Amyloid-like PNFs", the mobility of peptides after they self-assemble to form fibers in solution may be different depending on the peptide-peptide and peptide-solvent interactions. This, in turn, can potentially have consequences on the stability of fibers, their interaction with surroundings, and biological activity, such as retroviral infectivity enhancement. We quantified the mobility of peptides by calculating the root mean square fluctuation (RMSF) of each residue from our coarse-grained simulations. For all peptides, RMSF of amino acids in a single fiber in solution (Fig. S36A) is smaller and varies less along the peptide chain compared to the RMSF of amino acids when fibers are interacting in solution (Fig. S36B). Interestingly, for peptides with favorable fiber-fiber interaction, we observe an increase in the motion of the residues closer to the C-terminus, whereas the N-terminal amino acids in the peptide

sequence are more stationary. This result along with qualitative examination of simulation trajectories, show that even after the fibers have reached an equilibrium structure, i.e. the fiber-fiber distance does not change, there is considerable motion in the peptides. This result shows that the dynamic behavior of these supramolecular fibers are different when individual fibers aggregate in solution to form “bundles” as opposed to fibers in dilute solutions surrounded by solvent. Furthermore, large RMSF values are observed for fibers that do not aggregate in solution (e.g. CKIKQIINMWQ) or those that lose stability and remix to form new fibers (e.g. CKIKQII), but small RMSF values are found for those structures where fiber aggregation is the strongest (e.g. VHCVNITIK and KFKFQFNMWQ). Thus, when fibers aggregate to form hierarchical assemblies, the mobility of peptides that make up the supramolecular fiber is reduced.

But how does this dynamic behavior affect the stability of fibers? While the aggregated fibers remain stable in solution in our coarse-grained simulations with multiple interacting fibers, we do not observe any change in fiber morphologies over the 10 μs of simulation time. However, since these simulations are performed with coarse-grained models, we cannot make direct connections to their secondary structure or β-sheet content. Furthermore, we computed the RMSF from atomistic model of a single fiber in solution, both self-assembled and pre-formed, and the corresponding β-sheet formation in these structures (Fig. S37). We find that there is no correlation ($r^2 = 0.0055$ and 0.035 for self-assembled and pre-formed single fibers, respectively) between number of β-sheets and RMSF of peptides. It should be noted that this is only true for single fibers in solution. We do not know whether peptide mobility in aggregated fibers have different consequences on their β-sheet content. Larger scale atomistic simulations of aggregated fibers would need to be performed, which is something we would like to pursue in future studies.

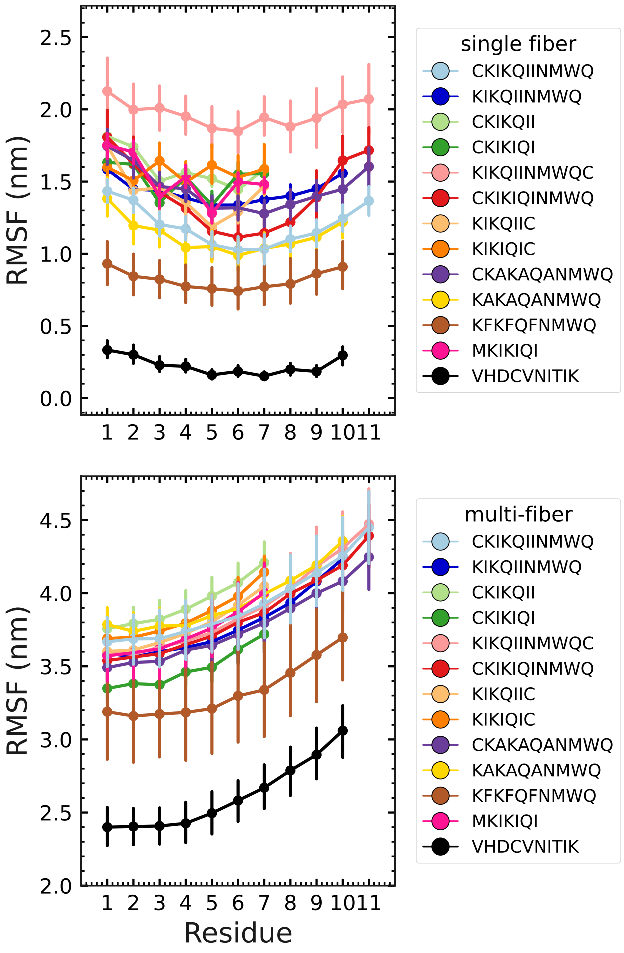

Figure S36: Average room mean square fluctuation (RMSF) for each residue calculated from coarse-grained single fiber (top) and multi-fiber (bottom) simulations. For single fiber the last 4 µs of 15 µs simulation, and for the multi-fiber system the last 2 µs of 10 µs simulation is used for averaging. Error bars are calculated from the standard deviation between different peptides in the system.

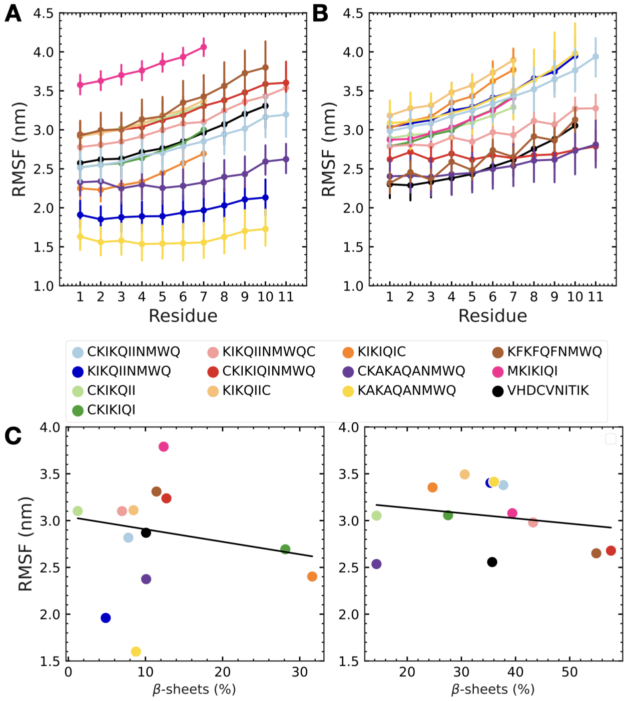

Figure S37: Average room mean square fluctuation (RMSF) for each residue calculated from atomistic (A) self-assembled and (B) pre-formed single fiber simulations. For both, the 100 ns of simulation is used for averaging. (C) Average RMSF for each peptide chain is plotted against average percentage of β-sheets calculated from self-assembled (left) and pre-formed (right) single fiber simulations, respectively.

References

(S1) Best, R. B.; Zhu, X.; Shim, J.; Lopes, P. E. M.; Mittal, J.; Feig, M.; MacKerell, A. D. J. Optimization of the Additive CHARMM All-Atom Protein Force Field Targeting Improved Sampling of the Backbone ϕ, ψ and Side-Chain χ^1 and χ^2 Dihedral Angles. *Journal of Chemical Theory and Computation* **2012**, *8*, 3257–3273, PMID: 23341755.

(S2) Jorgensen, W. L.; Chandrasekhar, J.; Madura, J. D.; Impey, R. W.; Klein, M. L. Comparison of Simple Potential Functions for Simulating Liquid Water. *J. Chem. Phys.* **1983**, *79*, 926–935.

(S3) Darden, T.; York, D.; Pedersen, L. Particle Mesh Ewald - an N.Log(N) Method for Ewald Sums in Large Systems. *J. Chem. Phys.* **1993**, *98*, 10089–10092.

(S4) Hess, B.; Bekker, H.; Berendsen, H. J. C.; Fraaije, J. G. E. M. LINCS: A linear constraint solver for molecular simulations. *Journal of Computational Chemistry* **1997**, *18*, 1463–1472.

(S5) Frishman, D.; Argos, P. Knowledge-based protein secondary structure assignment. *Proteins: Structure, Function, and Bioinformatics* **1995**, *23*, 566–579.

(S6) Humphrey, W.; Dalke, A.; Schulten, K. VMD – Visual Molecular Dynamics. *Journal of Molecular Graphics* **1996**, *14*, 33–38.

(S7) Wassenaar, T. A.; Pluhackova, K.; Böckmann, R. A.; Marrink, S. J.; Tieleman, D. P. Going Backward: A Flexible Geometric Approach to Reverse Transformation from Coarse Grained to Atomistic Models. *Journal of Chemical Theory and Computation* **2014**, *10*, 676–690, PMID: 26580045.

(S8) Álvarez, Z.; Kolberg-Edelbrock, A. N.; Sasselli, I. R.; Ortega, J. A.; Qiu, R.; Syrgiannis, Z.; Mirau, P. A.; Chen, F.; Chin, S. M.; Weigand, S.; Kiskinis, E.; Stupp, S. I.

Bioactive scaffolds with enhanced supramolecular motion promote recovery from spinal cord injury. *Science* **2021**, *374*, 848–856.

(S9) Sasselli, I. R.; Syrgiannis, Z.; Sather, N. A.; Palmer, L. C.; Stupp, S. I. Modeling Interactions within and between Peptide Amphiphile Supramolecular Filaments. *The Journal of Physical Chemistry B* **2022**, *126*, 650–659, PMID: 35029997.

(S10) Kaygisiz, K.; Rauch-Wirth, L.; Dutta, A.; Yu, X.; Nagata, Y.; Bereau, T.; Münch, J.; Synatschke, C. V.; Weil, T. Data-mining unveils structure–property–activity correlation of viral infectivity enhancing self-assembling peptides. *Nature Communications* **2023**, *14*, 5121.
